# Supplementary material for: Effectiveness of graded motor imagery protocol in phantom limb pain in amputed patient: Protocol of a randomized clinical trial
Source: PLoS One. 2022 Aug 25;17(8):e0273356. doi: 10.1371/journal.pone.0273356 (PMC9409541; doi:10.1371/journal.pone.0273356)
Supplement: S2 File — (DOCX) [file pone.0273356.s002.docx]

**RESEARCH PROJECT**

**Names and surnames of the principal researcher:**

Sandra Rierola Fochs

**Thesis supervisors**

Eduard Minobes Molina

Jose Antonio Merchán Baeza

**Research group**

Research Group on Methodology, Methods, Models and Outcomes of Health and Social Sciences (M3O) .Universitat de Vic-Universitat Central de Catalunya (UVic-UCC).

**Date and place**

Vic, January 10, 2022

**Title of the doctoral thesis project**

Effectiveness of graded motor imagery in phantom limb pain in amputated patient.

**INTRODUCTION**

The study presented below is part of a doctoral thesis, which consists of four differentiated and longitudinal parts over time. Each of the parts is related to one of the objectives set out in the thesis

| Objectives | Type of study |
| --- | --- |
| 1. Develop a graded motor imagery (GMI) protocol for a patient with phantom limb pain (PLP) after an amputation. | Systematic review |
| 1. Validate the developed protocol. | Study with Delphi methodology |
| 1. Analyze the effectiveness of the protocol in this patient profile. | Randomized clinical trial |
| 1. Know the experience of living with PLP and identify the satisfaction obtained with the intervention. | Phenomenological study |

The first part of the thesis consisted of a systematic review. Its purpose was to analyze the effectiveness of the GMI and each of the techniques that make it up (lateral recognition, motor imagery and mirror therapy) in the amputee patient. This review made it possible to analyze the details of the intervention used (intensity, frequency, duration) in each of the techniques in order to later elaborate the protocol. Once the protocol was designed, the second part of the thesis consisted of its validation. A group of national and international experts in the field of neurorehabilitation and / or pain validated the protocol through a study with Delphi methodology. This second study validated the GMI protocol called the GraMI protocol. These first two parts of the thesis were passed by the ethics committee of the University of Vic-Universitat Central de Catalunya (UVic-UCC) on January 25, 2021 with a favorable opinion with code 143/2021.

We are currently in the third part of the thesis, which aims to conduct a randomized clinical trial (RCT) to analyze the effectiveness of the GraMI protocol on phantom limb pain, quality of life, functionality and associated psychological aspects. In parallel with the RCT, it is also intended to carry out the fourth part of the thesis, which will consist of a phenomenological study in order to know the experience of living with PLP and identify the satisfaction obtained with the intervention. These last parts have also passed the ethics committee of the University of Vic-Universitat Central de Catalunya on November 22, 2021 with a favorable opinion with the code 185/2021.

Below are all the aspects related to these last two studies.

1. **BACKGROUND:**

The International Association for the Study of Pain defines phantom limb pain (PLP) as that pain or sensation referred to in a part of the body that has been previously removed as if it were still present(1,2). It affects between 55-80% of the population who has suffered an amputation, but not in the same way, frequency and intensity, therefore, there is no single definition of PLP as each person perceives it differently(3). In most cases, it appears the week after the amputation, but it can also appear after a few weeks or even months. In many cases it can last for years and become a chronic pain(4). There are different theories that try to explain the neurological basis of phantom limb pain, but there is no one dominant theory over the others. It is known that after amputation there is an alteration that causes changes at the peripheral, spinal cord and central level and can be associated with psychological disorders, such as depression or anxiety, which can influence the chronicity of the pain and the quality of life of the person (1,5,6). The main hypothesis is that after amputation, maladaptive plasticity occurs in the primary motor and sensory cortex, causing an incongruity of information between visual, motor and sensory, responding to pain. Currently, there are different lines of treatment to try to lower PLP; pharmacological, surgical and physiotherapeutic therapy(3).

GMI is a form of physiotherapeutic treatment, progressive, designed to train and reorganizes the brain based on three consecutive and progressive techniques: restoration of laterality, motor imagery and mirror therapy(6). GMI is based on different neuroscientific foundations, such as neuroplasticity and the use of mirror neurons with the idea that the reorganization of the cerebral cortex could help reduce pain(5).

1. **JUSTIFICATION:**

There is scientific evidence of GMI in the field of chronic pain, especially in the area of ​​complex regional pain syndrome, which shows positive effects in reducing pain(7,8). However, little evidence is found on the implementation of GMI in the patient with PLP after amputation(9). As a result, there is a need to design and validate an GMI protocol to address the PLP and test its effectiveness. The aim of this protocol is to provide healthcare professionals with a standardization of the intervention.

For all this, at the beginning of the thesis a systematic review was carried out in order to know the effectiveness of the GMI, and the techniques that form it, on the PLP in the amputated patient, and thus, to be able to design a protocol. of IMG based on scientific evidence. The systematic review, carried out between December 2020 and January 2021, revealed the effectiveness of the three techniques that make up the GMI in the PLP, but in those studies where the combination of the three techniques was used together, it was more effective. This protocol was subsequently validated by a group of international experts through a study with the Delphi methodology, resulting in the GMI protocol called GraMI. This Delphi study was published in the journal IJERPH on November 30, 2021(10).

The GraMI protocol allows the patient to perform it autonomously and individually at home with the follow-up of a professional. It is necessary to have a mirror box and a mobile application to be able to carry out the intervention. Today, the majority of the population has access to new technologies, which generate more motivation, dynamism, adherence and continuity with the intervention compared to other techniques(8).

1. **HYPOTHESIS**

The GraMI protocol can have a positive effect on the decrease in PLP in the amputee patient, and as a result, it can decrease the associated psychological factors and improve the person's quality of life and functionality.

1. **OBJECTIVES**

**Main objective:**

- To study the effectiveness of the GraMI protocol on PLP in the amputee patient.

**Specific objectives:**

- Design a mobile application to be able to carry out the intervention and determine its usability.

- Analyze the effectiveness of the GraMI protocol in the quality of life, functionality and psychological aspects of amputated patients.

- Know the experience of the person with PLP after amputation and identify the satisfaction obtained with the intervention

1. **DESIGN**:

A RCT with a mixed methodology will be carried out, with two parallel arms and blindly simple, following the recommendations of *the Consolidate Standards of Reporting Trials - CONSORT*(11), and a phenomenological study through semi-structured interviews, following the recommendations of *the Standards for Reporting Qualitative Research*(7) which will be carried out once the intervention is over. The clinical trial was registered at ClinicalTrials.gorv (registration number: NCT05083611).

1. **SETTING:**

It will be done at the community level individually. The intervention will be carried out by the participant himself / herself and will be supervised with a follow-up by the principal researcher. The semi-structured interviews will be individual and carried out in person or by video call once the intervention is over.

1. **SIMPLE SIZE**

The sample was calculated using the G * Power program, taking into account an alpha error of 0.5, a beta error of 0.8, and an effect size of 0.81(9). In order to calculate the sample we rely on existing scientific evidence. A study11 has been selected to assess phantom limb pain using the McGill Pain Short-Form Questionnaire, and the intervention used is based on mirror neurons. The total number of participants would be 50, with 25 in each group.

1. **INCLUSION AND EXCLUSION CRITERIA:**

**Inclusion**:

- People over 18 years old.

- Amputation of a limb.

- Minimum score of 3 on the visual analogue pain scale (VAS).

- Under pharmacological treatment for pain

- Pharmacologically stable.

- Have hospital discharge.

**Exclusion**:

- Visual alterations (hemianopsia)

- Significant neurological or cognitive impairments.

- Have previously received treatment with IMG.

**Removal**:

- Changes in medications that directly affect the main variable pain.

- Changes in the use or not of prostheses during the intervention.

Currently, the main line of treatment is pharmacological. As a result, most participants will receive medical treatment. So that this is not a condition in analyzing the effectiveness of our intervention, it will be analyzed that the participants are pharmacologically stable. By pharmacologically stable we mean that during the intervention there can be no changes in the drug that directly influences the PLP. There may also be no modifications to medications prescribed for other aspects but which may condition the PLP. An example of the latter would be antidepressants, which are adjuncts to the medications provided by PLP. If there are changes in medication that do not affect the pain, the intervention will be continued.

Prior to the intervention, the participants must have the established dose of medication, usually this dose is administered gradually until stability is achieved. Therefore, medication will be one of the variables to be collected to analyze and monitor this stability.

On the other hand, the elimination criterion according to the usefulness of the prosthesis is based on the fact that if the participant is wearing prosthesis, he / she will use it during the whole intervention, and I live in reverse, since the prosthesis could be a conditioning factor to study the effectiveness of our intervention.

1. **PARTICIPANTS:**

Participants will be recruited through the reference health professional of different hospitals that have amputee units, including the Hospital de la Santa Creu de Vic, Hospital de Sant Jaume de Manlleu, Hospital Pere Virgili, Asepeyo Sant Cugat Catalonia (Spain), which will assess its eligibility according to the established inclusion criteria. Once identified, the possible participants will be notified to the principal researcher, who will contact them by telephone to offer them the opportunity to participate and send them, via online, the information sheet with the details of the study. Those who agree to participate must sign the informed consent form and be informed that they have a waiver form so that they can withdraw from the study at any time they wish. Participants will be able to start the intervention once they have been registered or are already at home.

1. **TYPES OF SAMPLING AND RANDOMIZATION:**

A non-probabilistic sampling rate type will be used. It consists of first determining a number of subjects with certain characteristics (inclusion criteria), and then selecting the first individuals who possess these characteristics and agree to participate in the study.

As participants are recruited, an outside person will randomize them through sealed envelopes that they will assign to the control or intervention group. An external person will perform the randomization so as not to influence the assignment of the participants. During the study, the only people who will be blinded will be the evaluators so that they do not have an influence on the assessments made according to the group to which they belong. Due to the nature of the intervention, and given that the information sheet explains the characteristics of the interventions, there is no guarantee that the participants will not deduce which group they belong to. However, the therapists who will perform the intervention cannot be blinded either.

1. **PROCEDURE:**

**Step 1: Design the application**

In order to be able to carry out the first two techniques of the GraMI protocol (laterality recognition and motor imagery) a mobile application is required. This application will be designed by a team of UVic-UCC engineers and will be available for all devices. It will only be used by the participants in the experimental / intervention group. If, at the end of the study, the hypothesis that the GraMI protocol is effective in reducing the PLP is verified, the participants in the control group will be given the opportunity to use the application to carry out the protocol. The estimated design time for the app is four months (November 2021-February 2022), which will include piloting to test its usability and accessibility. During its design, a gamified application will be developed to encourage the motivation and adherence of the participant and aspects will be defined that will help the principal investigator in the supervision and follow-up of the intervention. In addition, the following aspects will be taken into account:

- The principal researcher of the project will install the application on the mobile device of the participant during the first educational session. This will allow you to learn how it works and help you become familiar with the application. Upon entering the application, the participant must register with their full name, username and be asked for the level of amputation (top or bottom) and side (right or left).

- The application will have two image banks differentiated according to the level of amputation (upper or lower extremity). Depending on the selection previously made by the participant, only the image bank related to his / her amputation will appear.

- Once you have entered the application, you will receive a calendar with a 6-week duration of the intervention (3 weeks for the laterality recognition phase and 3 weeks for the motor image phase).

- Through lock screens you will only be able to access the technique and level of progression that touches you at each moment of the intervention. The activity to be performed will be activated each day and the previous activity will be blocked. In this way we can guarantee the intensity and frequency established by the protocol.

- Scheduled to provide a daily reminder notification. In addition, the principal researcher of the project will contact the participant by telephone to encourage continuity with the intervention.

- The application will be designed to encourage the motivation of the participant through rankings and dynamization. The aim is to achieve greater adherence and follow-up of the intervention.

- Each attempt will be registered in the application in a database that will only have access to the research group formed by Sandra Rierola, Eduard Minobes Molina and Jose Antonio Merchán Baeza. The information that will be recorded will be whether or not you have completed the activity, the number of hits with respect to the total number of attempts and the speed of reaction to answer each of the images. Once the intervention is over, this data will be extracted and imported into a database that will be stored by the participant and only and will have access to the research group. The purpose of this data is to observe the follow-up carried out by the participant during the intervention, the evolution he / she has made and to observe the response time used in the laterality recognition phase.

**Step 2: RCT**

The RCT description was performed following the *Templeate for Intervention Description and Replication (TiDier)* guide 26 in order to adequately describe all aspects of the intervention and promote study replication.

The duration of the intervention, both in the control group and in the intervention group, will be 9 weeks plus 12 weeks of follow-up. The estimated start date for recruitment will be March 2022 and will run until March-April 2023.

Both the participants in the control group and the intervention group will initially receive an educational session, in person or online, on the physiology of PLP, brain plasticity and the importance of rehabilitation. It will last approximately 30 minutes where they will be explained in detail that after an amputation there is an incongruity of visual, sensitive and motor information and maladaptive plasticity due to adjacent areas occupying the areas injured by the amputation. . This educational session will be given by the principal investigator of the project, who has clinical experience, teaching and research in the field of neurorehabilitation and pain.

Control group intervention:

After the educational session, the participant will continue with the conventional treatment he / she is receiving at that time. Participants will be provided with a follow-up diary in which they will have to record their activities, specifying the nature, frequency and duration of each activity.

At the end of the study, if the hypothesis that the GraMI protocol is effective in reducing the PLP is verified, the option will be given to the participants in the control group who can perform the intervention.

Intervention / experimental group intervention:

Participants in the intervention group will receive conventional treatment plus the GraMI protocol. If they do not receive conventional treatment, they will only perform the GraMI protocol.

GraMI protocol:

Education:

At the beginning of each of the three phases, there will be an educational session of approximately 30 minutes. During each of the sessions, online or in person, the purpose of the phase in which it is located will be explained, the procedure to be followed, common objectives will be established for each phase and the normal parameters to which must be reached in the laterality recognition phase:

- Accuracy of more than 80% of accurate images.
- Average image recognition speed.

In addition, during the first educational session, the operation of the application will be explained so that the participant can become familiar and ask any questions, and notifications will also be set up to generate a daily reminder.

It is divided into three educational sessions so as not to saturate the participant with information, in this way it will be possible to strengthen the follow-up of the intervention and to resolve any doubts that may arise. These sessions will be conducted by the principal investigator.

The three phases described below do not require that the physiotherapist be always present during the operation. The participant can carry out the intervention autonomously at home, with prior training. The mobile application will record when the intervention has been carried out in order to facilitate follow-up and once a week, the principal investigator will follow up by telephone with the participant to make sure that the intervention is being carried out. . In addition, the application will include the personal email and telephone number of the researcher in order to facilitate contact at any time.

Phase 1. Laterality recognition:

The participant must observe images shown in the application, depending on the level of injury will observe images of upper or lower extremities, and differentiate with the shortest possible time if it is the right or left side of the body.

- Session intensity: 10 minutes.
- Frequency: Two sessions a day.
- Phase duration: 5 days a week for three weeks.
- Phase progression:

Day 1-5: Images in neutral positions in the different planes.

Day 6-10: Images in different positions in the different planes.

Day 11-15: Interaction with objects and functional activities.

The accuracy of the accuracy of each activity and the time used to identify each image will be recorded through the application.

Phase 2. Motor imagery:

This phase will be done through the mobile application. During the activity, images will appear, depending on the level of amputation, and the participant will have to imagine that he / she places his / her limb in the same position as the one in the image and returns it to the initial position, without perform voluntary contraction, will only perform a mental simulation, whether it is the side of the amputation or not.

- Session intensity: 10 minutes.
- Frequency: Two sessions a day.
- Phase duration: 5 days a week for three weeks.
- Phase progression (Same progression as phase 1)

Day 1-5: Images in neutral positions in the different planes.

Day 6-10: Images in different positions in the different planes.

Day 11-15: Interaction with objects and functional activities.

Each activity will also be recorded through the app. Before the start of the motor imaging phase, during the educational session, the project researcher will assess the person’s ability to imagine through the kinesthetic and visual imagination questionnaire (KVIQ) 12. It is a 10-item quiz where the physiotherapist teaches different movements and the person has to rate their ability to imagine them in the first person. In the event of a impairment of imagination, the person will be taught different strategies to do so. One of them will be before the intervention to show him/her videos and images of the affected limb in order to pre-activate the areas through the observation of the action. Another strategy is to make your imagination run wild first or try to find a factor or situation that helps you to imagine it. On the other hand, pain can occur during the imagination phase. If it appears, the person can be instructed to first imagine the movement with the healthy limb. If the pain is still triggered, we can ask you to imagine the movement in the third person. These strategies will be modified throughout the intervention to allow the person to realize the mental imagination in the first person.

Phase 3. Mirror therapy:

This phase is done with a mirror box, the mobile application is no longer used. The mirror box will be provided by the research team and once the intervention is completed, it must be returned. The participant should place the mirror in the sagittal plane between the two extremities, so that he observes the healthy end in the mirror (reflection) and the amputated end is behind the mirror. The limb behind the mirror should initially have no voluntary contraction or intentional contraction. The participant will later be asked if he or she intends to contract the stump. The size of the mirror will vary depending on the level of injury and the joints involved. If pain appears during the intervention, external factors can be sought to help tolerate the intervention (music, images ...). However, if the pain persists, it should be stopped at that time.

- Session intensity: 20 minutes
- Frequency: Once a day.
- Phase duration: 5 days a week for three weeks
- Phase progression:

Day 1-5: Analytical movements of the joints involved depending on the level of injury.

Day 6-10: Sensitive stimulation of the affected parts (textures, shapes, temperature, vibration).

Day 11-15: Interaction with functional objects and activities.

In order to follow up the intervention, the main researcher of the study will contact the participant, by telephone, once a week, in order to resolve possible doubts, clarifications or simply to remind the importance of follow-up of the intervention. In addition, the application will generate a daily notification in order to encourage continuity with the intervention.

**Step 3: Phenomenological study:**

In parallel with the RCT, as the participants in the experimental group complete the intervention, a qualitative phenomenological study will be performed. This type of study allows us to describe the experience of a person through semi-structured individual interviews(12). In addition, it allows to describe a phenomenon or an experience through the own people who suffer it (13).

Quantitative studies do not assess the experience of living with DMF and how it affects a person's quality of life. A systematic review of 2016 shows that there is little qualitative scientific evidence on a person's perception of PLP. Within the evidence we can observe studies that describe the psychological factors derived from PLP15 or the affect on the quality of life of people with amputation of the lower extremity(13). Therefore, there is a need to know the experience of people with PLP after an amputation and to identify the satisfaction obtained with the intervention.

This study will begin approximately in April 2022 and will last approximately until June 2023. During the study procedure, the qualitative study will be explained in person or by telephone to those people who are part of the intervention group. The purpose of the study and its methodology will be explained. Those who agree to participate will be sent, via email, the informed consent form and the information sheet. The appropriate number of people to participate is 15. This number is based on the scientific evidence considered appropriate taking into account the purpose of the research, the purpose of the analysis, the time and resources available(13). However, it is a forecast, it will be the saturation of data that will indicate the number of participants needed(14). Once the intervention is over, each person will be allowed to choose at what time and format (online or face-to-face) the interview was best for them. The importance of conducting the interview in person will be emphasized due to the importance of non-verbal communication between both the interviewer and the interviewee during the interview. The interview will follow a semi-structured structure based on a script but will be adapted as the interview progresses. It will consist of the following parts: demographic details, aspects related to amputation, experience of suffering from PLP and identification of the satisfaction obtained with the intervention. The approximate time for each interview will be 30-45 minutes.

The researcher conducting the interview will be an external person who has not performed the intervention or randomization, so as not to influence the questions and answers. This person has theoretical sensitivity about PLP and mirror therapy and this will make it easier to delve deeper into the topics during the interviews and better understand the experience. Subsequently, two members of the research group will perform the transcripts and analysis of the results. The aim of this triangulation is to achieve the highest possible objectivity in the results. The interviews will be recorded in voice and video format in order to be able to make a transcript as optimal as possible and will help not to lose the facial and body expressions of the participant. Only the research group and the researcher who conducted the interviews will have access to the recorded material. Each participant will have previously signed the consent to the image and voice rights. Once the study is complete, the recordings will be deleted. Once the transcripts have been completed, they will be sent to each participant to corroborate the information. This process will also be performed after the analysis of the results. You can see the consent form and the interview template in the appendices.

1. **VARIABLES:**

The assessments will be carried out by two people outside the intervention and the phenomenological study. These people will be two physiotherapists with clinical experience in the field of neurorehabilitation and / or pain, trained in research methods, calibrated with each other in order to minimize bias and will be blinded by the assignment of the groups. An initial assessment, a post-intervention assessment (9 weeks) and a follow-up assessment (12 weeks post-intervention) will be performed. The assessments will be carried out in the homes of the participants, in person and will last approximately 25-30 minutes.

The variables to be studied are differentiated between sociodemographic variables, amputation characteristics, independent variables and dependent variables. The first assessment will be the one that will last longer as all the variables will be collected, but the second and third assessment will last approximately 30 minutes as only the dependent variables will be collected.

- **Socio-demographic variables**; gender, age, marital status, height, level of education, level of physical activity, pathological and traumatic history, drugs administered and dose.

The collection of these variables will make it possible to describe the study sample and identify that the participants meet the inclusion criteria mentioned above. In this way, a sample that is as homogeneous as possible is obtained.

- **Amputation characteristics**: Cause of amputation, level of amputation, side, time since amputation, prosthetic wearer, number of hours the prosthesis is used.

- **Independent variables**: Treatment, control and intervention. All the information relevant to the conventional treatment of each of the participants will be collected.

**- Dependent variables:**

o Phantom limb pain: Short form McGill Pain Questionnaire. It is a scale that assesses pain from a quantitative and qualitative point of view. It consists of 15 pain descriptors, of which 11 are sensitive categories and 4 are affective. In addition, it contains an analog visual pain scale. It is self-answering and each descriptor has three columns (medium, moderate, severe), where the participant must mark the degree to which that descriptor persists in their pain. If you are not present, you can leave it blank. Columns are categorized as 0; no pain, 1; mig, 2; moderate, 3; severe. It takes between 2-5 minutes to be answered.

o Quality of life: EuroQol-5D-5L(15). It is a self-challenging scale consisting of 5 dimensions: mobility, self-care, regular activities, pain / discomfort and anxiety / depression. Each dimension has 5 levels: no problems, mild, moderate, severe and extreme problems. The participant is asked to indicate his / her state of health by ticking the box together with the most appropriate statement in each of the 5 dimensions. In addition, it contains a numerical scale from 0 to 100 to quantify the degree of health on the day of the assessment. It takes between 5-7 minutes to be answered.

o Functionality: Functional Independence Scale (FIM) (16). It is a scale built on 18 items within 6 areas of operation: personal care, sphincter control, mobility, gait, communication and social knowledge. The maximum score for each item is 7 and the minimum is 1. It takes 5 minutes to be answered.

o Psychological aspects: Beck depression inventory (17). It is a self-answering scale of 21 questions. Each question is quantified on a 4-point scale ranging from 0 to 3, with 0 experiencing no symptoms and 3 experiencing symptoms severely. It takes between 5-10min to be answered.

The following is a timetable detailing when each of the variables set out above will be collected.

| **Variable** | **Scale** | **Baseline** | **Post-intervention (9weeks)** | **Follow-up**  **(12 weeks post-intervention)** |
| --- | --- | --- | --- | --- |
| Socio-demographic |  |  |  |  |
| Amputation characteristics |  |  |  |  |
| Pain | SF-MPQ |  |  |  |
| Quality of life | EuroQol-5D-5L |  |  |  |
| Funcionality | FIM |  |  |  |
| Psychological aspects | Beck |  |  |  |

1. **STATISTICAL ANALYSIS:**

When performing a randomized cynical trial with mixed methodology we find two differentiated data analyzes.

Quantitative analysis:

The data obtained as study variables will be coded at the end of the collection, processed and analyzed by the members of the research group.

The population to be used as a sample in the analysis will include all participants who meet all the inclusion criteria, do not have any exclusion criteria and have given their consent to participate in the study. If a participant leaves before completing the study, he / she will not be replaced by another participant.

In the case of participants who have not completed the study due to dropout, have not fully completed treatment, or have even changed treatment, any data that may have been collected will be analyzed for intent to treat. The minimum number of sessions to be considered for the results will be 80%.

The program to be used for statistical analysis will be the SPSS5 program. A confidence level of 95%, and / or a statistical significance p <0.05 will be considered.

If a deviation from the intended method is considered necessary during the data analysis process, the reasons for any change will be fully documented.

To describe the sample, a descriptive statistic will be performed analyzing the mean, standard deviation, minimum, maximum and percentages of the sociodemographic variables. In addition, it will be possible to look at the average of those included and excluded in order to justify the excluded people. In this way it will be possible to describe the sample and observe the homogeneity between groups.

Next, a univariate and bivariate statistical analysis will be performed. First of all, it will be assessed whether or not the study follows a normal distribution in each of the variables. This analysis will determine whether to use parametric or non-parametric tests in the analysis of results based on the "p" value.

Equality of variance will then be analyzed through the Levenne test and finally the results will be analyzed. There will be a pre- and post-intervention analysis between groups and intragroups with a three-month follow-up. We can also analyze possible correlations between variables.

Qualitative analysis:

Qualitative analysis is a non-linear, interactive and dynamic process. It will consist of different parts following Colaizzi’s 7-stage method (18). We will start by transcribing the interviews and familiarizing ourselves with the data, then coding those ideas that are related to the research question. The codes obtained will be grouped into subcategories that share common aspects and will be described and located in the phenomenon studied. Finally, these subcategories will be categorized by looking for general ideas and possible relationships. The analysis will be performed through the program Atlas.ti 8.0.

1. **ETHICAL ASPECTS**

Recommendations for Good Clinical Practice: This study is based on the recommendations of Good Clinical Practice, the Declaration of Helsinki of the World Medical Association (mentioned in the 64th General Assembly, Fortaleza, Brazil, October 2013) and the applicable legal regulations. Therefore, all the researchers involved will sign a certificate of having read and understood this statement. If necessary, an adverse event notification form will be included in the appendices. It is necessary to maintain a rigorous and continuous quality control, which can guarantee the accuracy and scientific rigor of the data obtained, maintaining the conditions of homogeneity during the process of collecting the information. If necessary, the creation of a logistics committee will allow the proper coordination of all study groups and work to contrast the scientific coordination, methodological advice and the quality of the information obtained.

Information to be provided to participants and type of consent to be requested in the study: The model information to be provided to participants and the type of informed consent to be requested will be specified in the annexes to this effect. The appendices will also include, if necessary, a waiver sheet to allow participants to leave the study at any time. All participants will be verbally informed during the process of inclusion in the study by one of the researchers and will be sent online the fact sheets with all the detailed aspects. Subsequently, they will be asked for informed consent. They will also be provided with the consent form on image and voice rights.

Confidentiality: the strictest rules of professional conduct and confidentiality must be maintained at all times, and compliance with Regulation 2016/679 of the European Parliament and of the Council of 27 April 2016 on the protection of individuals ( RGPD) and Organic Law 3/2018, of 5 December, on the Protection of Personal Data and the Guarantee of Digital Rights (LOPD-GDD). The participant's right to confidentiality is paramount. The identity of the participant in the study documents must be coded, and only authorized persons will have access to personally identifiable details in the event that the data verification procedures require the inspection of these details. Identifiable personal details must always be kept confidential and will only be accessed by the principal investigator, the promoter and the persons authorized by the principal investigator and the relevant Health Authorities. In relation to the phenomenological study, it will not be possible to encode the identity of the participants as during the transcription phase we will have the video of the participant. The person responsible for making the transcripts will at all times guarantee the confidentiality of the data and once the transcripts have been made the videos will be deleted.

We can see all these documents in the appendices.

1. **EXPECTED BENEFITS**

At the participant level, the GraMI protocol is expected to decrease the DMF and, as a result, improve the quality of life and reduce the associated psychological factors.

At the level of participating hospitals, it is expected that they will be able to know a standardized way of applying IMG in this profile of patients.

At the level of the research group, it is expected to make communications in conferences and publications on the protocol and the results obtained, as well as its dissemination and implementation. In addition, the aim is to advance the doctoral thesis and try to get funded projects to continue research in this field.

1. **POSSIBLE UNWANTED OR SIDE EFFECTS**

The intervention is not invasive, so no serious side effects are expected with the intervention.

1. **COMPENSATION OR INSURANCE FOR PARTICIPANTS**

There is no consideration or insurance for the participants. If they wish, evolution reports can be prepared, comparing the initial, final and follow-up status of each participant.

**Participant Fact Sheet:**

Some of the members of the research team Research Group on Methodology, Methods, Models and Outcomes of Health and Social Sciences (M3O) of the University of Vic-Universitat Central de Catalunya (UVic-UCC), we carry out the project of Research "Effects of graduated motor image on phantom limb pain in the amputee patient". The principal researcher of this project is Sandra Rierola Fochs.

First, the research group developed a protocol for the intervention of the graded motor imagery in the amputee patient. This protocol has been validated by a committee of experts on the subject, reaching the final protocol (GraMI protocol). Second, it seeks to assess the effectiveness of the protocol obtained. Therefore, it is relevant to conduct an intervention study. The following centers are participating in the project: University of Vic, Hospital de la Santa Creu de Vic, Hospital Pere Virgili, Asepeyo Sant Cugat.

Study participants will be divided into two groups at random, one group will be called control and the other group will be called intervention. The intervention for both groups will last 9 weeks. The participants who are part of the control group will perform the treatment that is performed in a protocol manner in the center / hospital, while the participants who are part of the intervention group will perform the GraMI protocol. During this time, three assessments will be made, one before the start of treatment, the second after the end of treatment (9 weeks) and the third 12 weeks after the intervention (follow-up). The variables to be assessed are phantom limb pain, quality of life, functionality and psychological aspects. It will be assessed through validated scales. In addition, participants assigned to the intervention group will be offered to participate in another study that will consist of a semi-structured individual interview in order to understand how PLP affects the quality of life of those who suffer from it and identify satisfaction obtained with the intervention. Interviews will be video and voice recorded for later analysis and will last approximately 30-45 minutes. Once the study is complete, the recordings will be deleted.

The expected benefits of participating in the study are a possible reduction in phantom limb pain, leading to an improvement in quality of life.

There are no risks to your participation.

In the context of this research, we ask for your collaboration to include it in the group of people who will participate in the project, as you meet the following inclusion criteria:

- Adult over 18 years.

- Amputation of a limb.

- Minimum pain of 3 on the analog visual pain scale.

- Pharmacologically stable

- Have hospital discharge.

This collaboration is voluntary, has no cost to participants and involves cooperation in:

- 3 educational sessions of 30 minutes each session where the procedure of each of the phases of the intervention will be explained and possible doubts will be able to be solved. There will be a session every two weeks (online / face-to-face)

- Perform an intervention of 10-15 minutes, twice a day, 5 days a week (rest weekend), over 9 weeks.

- There will be 3 assessments during the study, an initial assessment at the beginning, a second assessment at the end of the intervention (9 weeks), and a third and final assessment three months after the end of the intervention.

All participants will be assigned a code by which it is impossible to identify the participant with the answers given, fully guaranteeing confidentiality. The data obtained from your participation will not be used for any purpose other than that explained in this information sheet and will become part of a data file for which the principal investigator will be responsible and only and will have access to it, and the other members of the investigation, Eduard Minobes Molina and Jose Antonio Merchán Baeza.

At the end of the study, you will be able to request information on the results from the principal investigator if you wish, as well as receive an initial and final report of the assessments made.

The study data file will be under the responsibility of the principal investigator, before whom she will be able to exercise at all times the rights established by Organic Law 3/2018, of 5 December, on the Protection of Personal Data and the guarantee of rights. and General Regulation (EU) 2016/679 of 27 April 2016 on data protection and supplementary regulations.

We are at your disposal to resolve any questions. You can contact the principal investigator via the email address: [sandra.rierola@uvic.cat](mailto:sandra.rierola@uvic.cat)

**Informed consent form:**

I, (name and surname) ___________________________________________, of legal age, with DNI ___________________, acting in my own name and interest.

I DECLARE THAT:

I have received information about the "Effects of Graded Motor Imagery on Phantom Limb Pain in Amputated Patient" project, of which I have been provided with the fact sheet attached to this consent and for which I am requesting my participation. I understood the meaning, my doubts were clarified and the actions of the study were explained to me. I have been informed of all aspects related to the confidentiality and protection of data with regard to the management of personal data involved in the project and the guarantees given in compliance with Organic Law 3/2018, of 5 December, of protection of personal data and guarantee of digital rights and General Regulation (EU) 2016/679 of 27 April 2016 on data protection and supplementary regulations.

My collaboration in the project is entirely voluntary and I have the right to withdraw at any time, and revoke this consent, without this being able to adversely affect me. In the event of withdrawal, I have the right to have my data deleted from the study file.

For all this,

I GIVE MY CONSENT TO:

1. Participate in the project "Effects of graded motor imagery on phantom limb pain in the amputated patient"

2. That the Research Group on Methodology, Methods, Models and Outcomes of Health and Social Sciences (M3O) and the researcher Sandra Rierola Fochs as IP, can manage my personal data and disseminate the information that the project will generate. It is guaranteed that my identity and privacy will be preserved at all times, with the guarantees established in Organic Law 3/2018, of 5 December, on the Protection of Personal Data and the guarantee of digital rights and the General Regulation (EU) 2016 / 679, of 27 of 27 April 2016, on data protection and complementary regulations.

3. That the Research Group on Methodology, Methods, Models and Outcomes of Health and Social Sciences (M3O) team keep all records made about me in electronic form, with the guarantees and terms legally provided, for the time that was necessary to fulfill the functions of the project.

City, day / month / year

___________________________ ___________________________

Participant Signature IP Signature

**Information sheet aimed at participants on conducting individual interviews:**

Dear participant,

Researchers who are part of the research group in Methodology, Methods, Models and Results of Health and Social Sciences (M3O) of the University of Vic- Central University of Catalonia (UVic-UCC) are carrying out a scientific research entitled “Effects of the graded motor imagery on the pain of the phantom limb in the amputated patient”.

The first (quantitative) part of this project is already under development. For the second part (qualitative), we want to understand how phantom limb pain (PLP) affects the quality of life of people who suffer from it and identify the satisfaction obtained with the intervention.

Conducting individual interviews is considered the best way to delve into an experience and understand a phenomenon from all its perspectives, so we would love to hear deeply about your experience and experience.

We offer you two different ways to conduct the interview, via the Internet using a Zoom program or in person. The interview is expected to last approximately 30-45 minutes.

We want the participant to have the flexibility to decide the date and time they may be available to participate in the interview. Therefore, the researchers who will participate in the interview will try as much as possible to adjust their schedule according to their availability.

During the interview, in the case of the Internet, we recommend that you be alone in a room or office without distractions, such as other people's conversations, excessive environmental noise, etc. Researchers will also be alone in a room or office to make sure no one else can hear the interview or will use headphones when this condition cannot be met during business hours.

To perform the data analysis of this research, we need to write down everything that is said during the interview, for this reason, we need to record the session so that we can then transcribe the entire interview word for word. Once all the content has been transcribed, your recording and written transcript will be emailed to you and you will be asked to confirm the veracity.

Once the veracity of the transcript is confirmed, the recording will be permanently deleted. The text of the conversation will be stored in a password-protected folder and will not be accessible to third parties.

If you have any questions or need any clarification regarding the conduct of the interview, please do not hesitate to contact the researcher in charge of the interviews Sandra Rierola (630841957, sandra.rierola@uvic.cat).

Once again, we thank you for your contribution to this scientific research as a participant. Yours faithfully.

**Consent for audiovisual recording**

Researcher: Sandra Rierola Fochs

Participant:

CONSENT: I ​​agree with:

I accept delivery to record in audiovisual support.

I understand and understand that recording is related to promoting knowledge about phantom limb pain and identifying satisfaction with the intervention. It will be used exclusively for research, anonymity. (Organic Law 15/1999 of 13 December, on the Protection of Personal Data, Royal Decree 994/1999, of 11 June). Access to the data will be restricted to authorized personnel. The storage of the recording will comply with all the necessary security measures to comply with the aforementioned law.

I understand that I may withdraw my consent at any time, and that this will not affect the treatment I receive so far or in the future.

Participant's signature:

Date:

Informant Signature:

Date:

**Waiver sheet**

Study title: Efficacy of graduated motor image in phantom limb pain in amputee patient

Jo, ................................................ .................................................. ................................., with the DNI nº ......................................, I declare that my wish is to leave the studio in which I have been participating.

I declare that there was no pressure or insistence on giving my personal reasons for the waiver, in accordance with the rules and procedures for inclusion in the study.

I spoke to: Sandra Rierola Fochs

I understand that my participation was voluntary at all times and it was my decision to leave the studio.

I understand that I can retire from the studio:

1 ° Without having to give any explanation, i

2 ° Without this fact being able to affect my relationship with the study researcher or the promoters.

Therefore, I renounce to continue participating in this study.

Signed:

Name and surname of the participant: …………… ... ………………………………………… ..

D.N.I .: …………………………… Age: ………… .. Date: ……………… ..

Signature of the principal investigator:

Principal Investigator: Sandra Rierola Fochs

Date: ..

**REFERENCES:**

1. Aiyer R, Barkin RL, Bhatia A, Gungor S. A systematic review on the treatment of phantom limb pain with spinal cord stimulation [Internet]. Vol. 7, Pain management. Future Medicine Ltd London, UK ; 2017 [cited 2021 May 10]. p. 59–69. Available from: https://www.futuremedicine.com/doi/abs/10.2217/pmt-2016-0041

2. Fehlings MG, Sarhane KA, Wilson J, Tsao JW, Sb F, Bn P, et al. a randomized, Controlled Trial of Mirror Therapy for Upper Extre1. Fehlings MG, Sarhane KA, Wilson J, Tsao JW, Sb F, Bn P, et al. a randomized, Controlled Trial of Mirror Therapy for Upper Extremity Phantom limb Pain in Male amputees. 2017 [cited 2021 May. 2017 [cited 2021 May 10];8. Available from: www.frontiersin.org

3. Collins KL, Russell HG, Schumacher PJ, Robinson-Freeman KE, O’Conor EC, Gibney KD, et al. A review of current theories and treatments for phantom limb pain. J Clin Invest [Internet]. 2018;128(6):2168–76. Available from: https://www.scopus.com/inward/record.uri?eid=2-s2.0-85048300573&doi=10.1172%2FJCI94003&partnerID=40&md5=db162b3f803316198bdb441e31992132

4. Vannuccini S, Petraglia F. Recent advances in understanding and managing phantom limb pain. F1000Research. 2019;8:1–11.

5. Osinski T. Imaginería motora graduada. Colloids Surfaces A Physicochem Eng Asp. 2019;39(2):1–10.

6. Mg DF, Manual T, Fisioterapeuta O. Imaginería motora graduada en el síndrome de miembro fantasma con dolor. 2012;19(4):209–16.

7. O’Brien BC, Harris IB, Beckman TJ, Reed DA, Cook DA. Standards for reporting qualitative research: A synthesis of recommendations. Acad Med. 2014;89(9):1245–51.

8. Ebrahimi N, Rojhani-Shirazi Z, Yoosefinejad AK, Nami M. The effects of virtual reality training on clinical indices and brain mapping of women with patellofemoral pain: a randomized clinical trial. BMC Musculoskelet Disord [Internet]. 2021;22(1):1–10. Available from: https://doi.org/10.1186/s12891-021-04785-6

9. Osumi M, Ichinose A, Sumitani M, Wake N, Sano Y, Yozu A, et al. Restoring movement representation and alleviating phantom limb pain through short-term neurorehabilitation with a virtual reality system. Eur J Pain (United Kingdom). 2017;21(1):140–7.

10. Rierola-fochs S, Adriana L, Minobes-molina E, Antonio J. Development and Validation of a Graded Motor Imagery Intervention for Phantom Limb Pain in Patients with Amputations ( GraMI Protocol ): A Delphi Study. 2021;

11. Moher D, Hopewell S, Schulz KF, Montori V, Gøtzsche PC, Devereaux PJ, et al. CONSORT 2010 explanation and elaboration: Updated guidelines for reporting parallel group randomised trials. Int J Surg [Internet]. 2012;10(1):28–55. Available from: http://dx.doi.org/10.1016/j.ijsu.2011.10.001

12. Lambert C. Edmund Husserl: la idea de la fenomenología. Teol y vida. 2006;47(4):517–30.

13. Trevelyan EG, Turner WA, Robinson N. Perceptions of phantom limb pain in lower limb amputees and its effect on quality of life: A qualitative study. Br J Pain [Internet]. 2016;10(2):70–7. Available from: https://www.scopus.com/inward/record.uri?eid=2-s2.0-84981363485&doi=10.1177%2F2049463715590884&partnerID=40&md5=fe9e7657d756d8c8478e29a0a6423882

14. Nuevos avances en la investigación social. La investigación social de segundo orden. 1998;

15. Herdman M, Gudex C, Lloyd A, Janssen M, Kind P, Parkin D, et al. Development and preliminary testing of the new five-level version of EQ-5D (EQ-5D-5L). Qual Life Res. 2011;20(10):1727–36.

16. de Azevedo Alves GA, Martinez BP, Lunardi AC. Assessment of the measurement properties of the Brazilian versions of the Functional Status Score for the ICU and the Functional Independence Measure in critically ill patients in the intensive care unit. Rev Bras Ter Intensiva. 2019;31(4):521–8.

17. Beck AT, Ward CH, Mendelson M, Mock J, Erbaugh J. An Inventory for Measuring Depression, Archives of General Psychiatry, 4. Arch Gen Psychiatry. 1961;4(6):561–71.

18. Wirihana L, Welch A, Williamson M, Christensen M, Bakon S, Craft J. Using Colaizzi’s method of data analysis to explore the experiences of nurse academics teaching on satellite campuses. Nurse Res. 2018;25(4):30–4.
