# Supplementary material for: Effectiveness of graded motor imagery protocol in phantom limb pain in amputed patient: Protocol of a randomized clinical trial
Source: PLoS One. 2022 Aug 25;17(8):e0273356. doi: 10.1371/journal.pone.0273356 (PMC9409541; doi:10.1371/journal.pone.0273356)
Supplement: S3 File — (DOCX) [file pone.0273356.s003.docx]

**PROJECTE D’INVESTIGACIÓ:**

**Dades personals**

**Noms i cognoms investigadora principal:**

Sandra Rierola Fochs

**Directors de tesis**

Eduard Minobes Molina

Jose Antonio Merchán Baeza

**Grup d’investigació**

Research Group on Methodology, Methods, Models and Outcomes of Health and Social Sciences (M_3_O).Universitat de Vic-Universitat Central de Catalunya (UVic-UCC).

**Data i lloc**

Vic, 10 de Gener del 2022

**Títol del projecte de tesis doctoral**

Eficàcia de la imatge motora graduada en el dolor del membre fantasma en el pacient amputat.

**INTRODUCCIÓ**

L’estudi que es presenta a continuació forma part d’una tesis doctoral, la qual està formada per quatre parts diferenciades i longitudinals en el temps. Cada una de les parts va en relació amb un dels objectius establerts en la tesis.

| Objectiu | Tipus d’estudi |
| --- | --- |
| 1. Dissenyar un protocol d’imatge motora graduada (IMG) per al pacient amb dolor del membre fantasma (DMF) després d’una amputació. | Revisió sistemàtica |
| 1. Validar el protocol dissenyat. | Estudi amb metodologia Delphi. |
| 1. Analitzar l’efectivitat del protocol en aquest perfil de pacients. | Assaig clínic aleatoritzat. |
| 1. Conèixer l’experiència de viure amb DMF i identificar la satisfacció obtinguda amb la intervenció. | Estudi fenomenològic. |

La primera part de la tesis va consistir en la realització d’una revisió sistemàtica. La finalitat de la qual era analitzar l’efectivitat de la IMG i de cada una de les tècniques que la formen (reconeixement de la lateralitat, imatge motora i teràpia mirall) en el/la pacient amputat. Aquesta revisió va permetre analitzar els detalls d’intervenció utilitzats (intensitat, freqüència, duració) en cada una de les tècniques per tal d’elaborar posteriorment el protocol. Un cop dissenyat el protocol, la segona part de la tesis va consistir en la seva validació. Un grup d’experts/es, nacionals i internacionals, sobre l’àmbit de la neurorehabilitació i/o dolor van validar el protocol a través d’un estudi amb metodologia Delphi. D’aquest segon estudi en va sortir validat el protocol d’IMG anomenat protocol GraMI. Aquestes dues primeres parts de la tesis van passar el comitè d’ètica de la Universitat de Vic-Universitat Central de Catalunya (UVic-UCC) el 25 de gener del 2021 amb un dictamen favorable amb el codi 143/2021.

Actualment, ens trobem en la tercera part de la tesis, en la qual es pretén realitzar un assaig clínic aleatoritzat (ECA) per analitzar l’efectivitat del protocol GraMI en el dolor del membre fantasma, en la qualitat de vida, la funcionalitat i els aspectes psicològics associats. Paral·lelament a l’ECA, també es pretén dur a terme la quarta part de la tesis, que consistirà en un estudi fenomenològic per tal de conèixer l’experiència de viure amb DMF i identificar la satisfacció obtinguda amb la intervenció.

A continuació es detallen tots els aspectes relacionats amb aquests dos últims estudis.

1. **ANTECEDENTS**

L'Associació Internacional per l’Estudi del Dolor defineix el dolor del membre fantasma (DMF) com aquell dolor o sensació referit en una part del cos que ha estat extirpada anteriorment com si encara estigués present^1,2^. Afecta entre un 55-80% de la població que ha patit una amputació, però no de la mateixa forma, freqüència i intensitat, per tant, no hi ha una definició única de dolor del membre fantasma ja que cada persona el percep de forma diferent^3^. En la major part dels casos apareix a la setmana següent de l'amputació però també pot aparèixer al cap d’unes setmanes o fins i tot de mesos. En molts casos pot perdurar anys d'evolució i convertir-se en un dolor crònic^4^. Hi ha diferents teories que intenten explicar la base neurològica del dolor del membre fantasma, però no hi ha una teoria dominant sobre les altres. Es coneix que després de l’amputació es produeix una alteració que provoca canvis a nivell perifèric, medul·lar i central i que pot anar associat a alteracions psicològiques, com ara la depressió o ansietat, que poden influenciar en la cronicitat del dolor i en la qualitat de vida de la persona^1,5-7^. La hipòtesis principal és que després de l’amputació es produeix una plasticitat maladaptativa a nivell del còrtex motor i sensitiu primari provocant una incongruència d’informacions entre visual, motora i sensitiva donant com a resposta el dolor. Actualment, hi ha diferents línies de tractament per intentar disminuir el DMF; farmacològica, quirúrgica i fisioterapèutica^3,7^.

La IMG és una modalitat de tractament fisioterapèutic, progressiva, dissenyada per entrenar i reorganitzar el cervell basat en tres tècniques consecutives i progressives: restauració de la lateralitat, imatge motora i teràpia mirall^5^. La IMG es basa en diferents fonaments neurocientífics, com la neuroplasticitat i la utilització de les neurones mirall amb la idea de que la reorganització de l’escorça cerebral podria ajudar a disminuir el dolor^6^.

1. **JUSTIFICACIÓ**

Hi ha evidència científica de la IMG en l’àmbit del dolor crònic, sobretot a nivell del síndrome de dolor regional complex, que mostra efectes positius en la disminució del dolor^8,9,10^. No obstant això, es troba escassa evidència sobre la implementació de la IMG en el pacient amb DMF després d’una amputació^11^. Per aquest fet, genera la necessitat de dissenyar i validar un protocol d’IMG per abordar el DMF i contrastar la seva efectivitat. A través d’aquest protocol es pretén proporcionar als/les professionals sanitaris/es una estandardització de la intervenció.

Per tot això, al començament de la tesis es va realitzar una revisió sistemàtica per tal de conèixer l’efectivitat de la IMG, i les tècniques que la formen, sobre el DMF en el/la pacient amputat, i així, poder dissenyar un protocol d’IMG basat en la evidència científica. La revisió sistemàtica, realitzada entre el desembre 2020 i gener 2021 va permetre conèixer l’efectivitat de les tres tècniques que formen la IMG en el DMF, però en aquells estudis on s’utilitzava la combinació de les tres tècniques juntes es mostrava major efectivitat. Posteriorment, es va validar aquest protocol a través d’un grup d’experts/es internacionals mitjançant un estudi amb metodologia Delphi, donant lloc al protocol d’IMG anomenat GraMI.

EL protocol GraMI permet al/la pacient realitzar-lo de forma autònoma i individual en el seu domicili amb el seguiment d’un/una professional. És necessari disposar d’un mirror box i una aplicació mòbil per poder dur a terme la intervenció. Actualment, la majoria de la població disposa d’accés a les noves tecnologies, les quals, generen més motivació, dinamisme, adherència i continuïtat amb la intervenció en comparació a altres tècniques^7^.

1. **HIPÒTESIS**

El protocol GraMI pot tenir efectes positius en la disminució del DMF en el/la pacient amputat/da, i com a conseqüència, pot disminuir els factors psicològics associats i millorar la qualitat de vida i funcionalitat de la persona.

1. **OBJECTIUS**

**Objectiu principal:**

- Estudiar l'efectivitat del protocol GraMI sobre el DMF en el/la pacient amputat/da.

**Objectius específics:**

- Dissenyar una aplicació mòbil per poder dur a terme la intervenció i determinar la seva usabilitat.
- Analitzar l’efectivitat del protocol GraMI en la qualitat de vida, funcionalitat i aspectes psicològics dels/les pacients amputats/des.
- Conèixer la vivència de la persona amb DMF després de l’amputació i identificar la satisfacció obtinguda amb la intervenció.

1. **Disseny:**

Es durà a terme un ECA amb metodologia mixta, amb dos braços paral·lels i cegament simple, seguint les recomanacions of the Consolidate Standards of Reporting Trials – CONSORT^8^, i un estudi fenomenològic a través d’entrevistes semiestructurades, seguint les recomanacions the Standards for Reporting Qualitative Research^9^ les quals es realitzaran un cop acabada la intervenció. L’assaig clínic es va registrar en ClinicalTrials.gorv (número de registre: NCT05083611). Aquest estudi va ser aprovat, amb un dictamen favorable, pel comitè d’ètica de la Universitat de Vic el 24 de novembre del 2021 amb el codi 185/2021.

1. **Àmbit:**

Es realitzarà en l’àmbit comunitari de forma individual. La intervenció la realitzarà el/la propi/a participant de forma autònoma i serà supervisada amb un seguiment per part de la investigadora principal. Les entrevistes semiestructurades seran individuals i realitzades de forma presencial o a través de videotrucada una vegada acabada la intervenció.

1. **Càlcul de la mostra**

El càlcul de la mostra s’ha realitzat a través del programa G*Power, tenint en compte un error alfa del 0,5, un error beta de 0,8 i un tamany de l’efecte de 0,81^10^. Per tal de calcular la mostra ens basem en la evidència científica existent. S’ha seleccionat un estudi^10^ que valora el dolor del membre fantasma a través del qüestionari Short-Form McGill Pain Questionnaire i la intervenció utilitzada és basada en les neurones mirall. El número total de participants seria de 50, sent 25 en cada grup.

1. **Criteris inclusió i exclusió:**

Inclusió:

- Persones de més de 18 anys.
- Amputació d’una extremitat.
- Mínima puntuació de 3 en l’escala visual analògica del dolor (EVA).
- Farmacològicament estables.
- Tenir l’alta hospitalària.

Exclusió:

- Alteracions visuals (hemianopsia)
- Alteracions neurològiques o cognitives importants.
- Haver rebut prèviament tractament amb IMG.

Eliminació:

- Canvis en la medicació que influeixi directament sobre la variable principal dolor.
- Canvis en la utilització o no de pròtesis durant la intervenció.

Actualment, la principal línia de tractament és la farmacològica. Per aquest fet, la majoria dels/les participants es trobaran sota tractament mèdic. Perquè això no sigui un condicionant en analitzar l’efectivitat de la nostra intervenció, s’analitzarà que els/les participants estiguin farmacològicament estables. Per farmacològicament estables entenem que durant la intervenció no poden produir-se modificacions en el medicament que influeixi de forma directa en el DMF. Tampoc poden produir-se modificacions de medicaments prescrits per altres aspectes però que puguin condicionar el DMF. Un exemple d’aquest últim cas serien els antidepressius que són coadjuvants dels medicaments que es proporcionen pel DMF. Si es produeixen canvis de medicació que no tenen influència sobre el dolor es seguirà amb la intervenció.

Prèviament a la intervenció els/les participants han de tenir la dosis de medicament establerta, normalment aquesta dosis s’administra de forma progressiva fins assolir l’estabilitat. Per tant, la medicació serà una de les variables a recollir per analitzar i fer un seguiment d’aquesta estabilitat.

Per altra banda, el criteri d’eliminació segona la utilitat de pròtesis es basa en què si el/la participant és portador de pròtesis la utilitzi durant tota la intervenció, i visc eversa, ja que la pròtesis podria ser un factor condicionant per estudiar l’efectivitat de la nostra intervenció.

1. **Participants:**

Els/les participants es reclutaran a través del/la professional sanitari/a de referència de diferents hospitals que disposin d’unitats d’amputats, entre ells l’Hospital de la Santa Creu de Vic, l’Hospital de Sant Jaume de Manlleu o l’Asepeyo de Sant Cugat del Vallès, Catalunya (Espanya), el qual valorarà la seva elegibilitat segons els criteris d’inclusió establerts. Un cop identificats els/les possibles participants seran comunicats a la investigadora principal, la qual es posarà en contacte amb ells/elles, via telefònica, per oferir-los la possibilitat de participar i enviar-los, via online, la fulla d’informació amb els detalls de l’estudi. Aquelles persones que acceptin participar hauran de firmar el full de consentiment informat i se’ls informarà que disposen d’una fulla de renúncia per poder retirar-se de l’estudi en el moment que ho desitgin. Els/les participants podran començar la intervenció al ser donats d’alta o que ja estiguin al seu domicili.

1. **Tipus de mostreig i aleatorització:**

S’utilitzarà un tipus de mostreig no probabilístic per quotes. Consisteix en fixar prèviament un número de subjectes amb unes característiques determinades (criteris d’inclusió), i posteriorment, seleccionar als primers individus que posseeixin aquestes característiques i acceptin participar en l’estudi.

A mesura que es recluti als/les participants, una persona externa els/les aleatoritzarà a través de sobres tancats que els/les assignarà al grup control o intervenció. Realitzarà l’aleatorització una persona externa per tal de no influir en la assignació dels/les participants. Durant l’estudi les úniques persones que estaran cegades seran els/les avaluadors/es perquè no tinguin influència sobre les valoracions realitzades en funció del grup al qual pertanyen. Degut a la naturalesa de la intervenció, i donat que en la fulla d’informació s’expliquen les característiques de les intervencions, no es pot garantitzar que els/les participants no dedueixin a quin grup pertanyen. Tanmateix, els/les terapeutes que realitzaran la intervenció tampoc podran ser cegats/es.

1. **Procediment:**

**Etapa 1: Disseny de l’aplicació**

Per a poder dur a terme les dues primeres tècniques del protocol GraMI es requereix una aplicació mòbil. Aquesta aplicació serà dissenyada per un equip d’enginyers de la UVic-UCC i estarà disponible per a tots els dispositius. El temps estimat de disseny són quatre mesos (novembre del 2021-febrer del 2022), entre els quals inclourà un pilotatge per provar la seva usabilitat i accessibilitat. Durant el seu disseny es desenvoluparà una aplicació gamificada per fomentar la motivació i adherència del participant i es definiran aspectes que ajudin a la investigadora principal en la supervisió i seguiment de la intervenció. A més es tindran en compte els següents aspectes:

- Disposarà de dos bancs d’imatges diferenciats segons el nivell d’amputació (extremitat superior o inferior).
- El/la participant només podrà realitzar la intervenció amb la freqüència i intensitat establerta pel protocol.
- A través de pantalles de bloqueig només podrà accedir a la tècnica i nivell de progressió que li toca en cada moment de la intervenció. D’aquesta forma podem garantitzar la intensitat i freqüència establertes pel protocol.
- Es programarà perquè proporcioni una notificació de recordatori al dia.
- Es dissenyarà fomentant la motivació del/la participant a través de rànquings i dinamització de l’aplicació. D’aquesta forma es pretén aconseguir major adherència i seguiment de la intervenció.
- Quedarà registrada la data de realització per tal de facilitar el seguiment de la intervenció.

**Etapa 2: ECA**

La descripció de l’ECA s’ha realitzat seguint la guia *Templeate for Intervention Description and Replicaction (TiDier)*^26^ per tal de descriure adequadament tots els aspectes relacionats amb la intervenció i afavorir la replicació de l’estudi.

La duració de la intervenció, tant del grup control com del grup intervenció, serà de 9 setmanes més 12 setmanes de seguiment. La data estimada d’inici del reclutament serà el març de l’any 2022 i s’allargarà fins al març-abril de l’any 2023.

Tant els participants del grup control com del grup intervenció rebran inicialment una sessió educativa, en format presencial o online, sobre la fisiologia del DMF, la plasticitat cerebral i la importància de la rehabilitació. Tindrà una durada aproximada de 30 minuts on se’ls explicarà de forma detallada que després d’una amputació es produeix una incongruència d’informacions visuals, sensitives i motores i una plasticitat maladaptativa degut a que àrees adjacents ocupen les àrees lesionades per l'amputació. Aquesta sessió educativa serà impartida per part de la investigadora principal del projecte, la qual té experiència clínica, docent i investigadora en l’àmbit de la neurorehabilitació i dolor.

Intervenció del grup control:

Després de la sessió educativa, el/la participant seguirà amb el tractament convencional que està rebent en aquell moment. En cas de no rebre cap tractament, seguirà amb el tractament pautat pel centre hospitalari en el qual hagi estat amb la freqüència i intensitat habitual, ja sigui farmacològic o fisioterapèutic.

Un cop acabat l’estudi, si es verifica la hipòtesis que el protocol GraMI té efectivitat en la disminució del DMF, es donarà la opció als/les participants del grup control que puguin realitzar la intervenció.

Intervenció del grup intervenció/experimental:

Els participants del grup intervenció rebran el tractament convencional més el protocol GraMI. En cas de no rebre tractament convencional només realitzaran el protocol GraMI.

Protocol GraMI:

Educació:

Al inici de cada una de les tres fases, es farà una sessió educativa, d’ aproximadament 30 minuts. Durant cada una de les sessions, via online o presencial, s’explicarà la finalitat de la fase en la que es troba, el procediment a seguir, s’establiran uns objectius comuns per a cada fase i s’explicaran els paràmetres normals als quals s’ha d’arribar en la fase de reconeixement de la lateralitat:

- - Precisió superior al 80% de les imatges encertades.
  - Velocitat mitja de reconeixement d’imatges.

A més, durant la primera sessió educativa s’explicarà el funcionament de l’aplicació per tal de que el/la participant pugui familiaritzar-se i demanar possibles dubtes, també es configuraran les notificacions per tal de generar un recordatori al dia.

Es divideix en tres sessions educatives per no saturar al/la participant d’informació, d’aquesta forma es podrà potenciar el seguiment de la intervenció i anar resolent possibles dubtes que sorgeixin. Aquestes sessions seran realitzades per part de la investigadora principal.

Les tres fases descrites a continuació no requereixen que el/la fisioterapeuta estigui sempre present durant la realització de la intervenció. El/la participant pot realitzar la intervenció de forma autònoma en el seu domicili, amb un previ entrenament. L’aplicació mòbil registrarà quan s’ha fet la intervenció per tal de facilitar el seguiment i un cop a la setmana, es farà un seguiment telefònic per part de la investigadora principal amb el/la participant per assegurar-nos que està realitzant la intervenció. A més, en l’aplicació estarà escrit el correu personal i número de telèfon de la investigadora per tal de facilitar el contacte en qualsevol moment.

Fase 1. Reconeixement de la lateralitat:

El/la participant ha d’observar imatges mostrades en la aplicació, en funció del nivell de lesió observarà imatges d’extremitats superiors o inferiors, i diferenciar amb el menor temps possible si es tracta del costat dret o esquerre del cos.

- - Intensitat de la sessió: 10 minuts.
  - Freqüència: Dues sessions al dia.
  - Duració de la fase: 5 dies a la setmana al llarg de tres setmanes.
  - Progressió de la fase:

Dia 1-5: Imatges en posicions neutres en els diferents plans.

Dia 6-10: Imatges en diferents posicions en els diferents plans.

Dia 11-15: Imatges amb interacció d’objectes i activitats funcionals.

A través de l’aplicació quedarà registrat la precisió d’encert en cada activitat i el temps utilitzat en la identificació de cada imatge.

Fase 2. Imatge motora:

El/la participant haurà d’imaginar-se que col·loca la seva extremitat en la mateixa posició que la de la imatge i la torna a la posició inicial, sense realitzar contracció voluntària, només realitzarà una simulació mental, indiferentment que sigui el costat de l’amputació o no. Cada imatge es mostrarà durant 5 segons.

- - Intensitat de la sessió: 10 minuts.
  - Freqüència: Dues sessions al dia.
  - Duració de la fase: 5 dies a la setmana al llarg de tres setmanes.
  - Progressió de la fase (Mateixa progressió que la fase 1)

Dia 1-5: Imatges en posicions neutres en els diferents plans.

Dia 6-10: Imatges en diferents posicions en els diferents plans.

Dia 11-15: Imatges amb interacció d’objectes i activitats funcionals.

A través de l’aplicació també quedarà registrada cada activitat. Abans de començar la fase de imatge motora es valorarà la capacitat d’imaginació de la persona a través del qüestionari d’imaginació cinestèsica i visual (KVIQ)^32^. És un qüestionari format per 10 ítems on el fisioterapeuta ensenya diferents moviments i la persona ha de puntuar la capacitat que té d’imaginar-los en primera persona. En cas de que hi hagi una alteració en la capacitat d’imaginació s’ensenyaran diferents estratègies a la persona per poder-ho realitzar. Una d’elles serà prèviament a la intervenció ensenyar-li vídeos i imatges de l’extremitat afectada per tal de preactivar les àrees a través de la observació de l’acció. Una altra estratègia és realitzar la imaginació primer amb l’extremitat sana o intentar buscar algun factor o situació que ajudi a imaginar-lo. Per altra banda, durant la fase de imaginació pot produir-se dolor. Si apareix, se li pot indicar a la persona que s’imagini primer el moviment amb l’extremitat sana. Si tot i així es desencadena el dolor, li podem demanar que s’imagini el moviment en tercera persona. Aquestes estratègies s’aniran modificant al llarg de la intervenció per acabar aconseguint que la persona pugui realitzar la imaginació mental en primera persona.

Fase 3. Teràpia mirall:

Aquesta fase es realitza amb un mirror box, ja no s’utilitza la aplicació mòbil. El/la participant ha de col·locar el mirall en el pla sagital entre les dues extremitats, de forma que observi l’extremitat sana en el mirall (reflex) i l’extremitat amputada estigui darrere del mirall. L’extremitat que està darrere del mirall inicialment no ha de fer cap contracció voluntària ni intenció de contracció. Més endavant es demanarà al participant que si que realitzi la intenció de contracció del monyó. La grandària del mirall variarà en funció del nivell de lesió i de les articulacions involucrades. Si durant la intervenció apareix dolor es poden buscar factors externs que ajudin a tolerar la intervenció (música, imatges...). Si tot i així, el dolor persisteix s’haurà d’aturar en aquell moment.

- - Intensitat de la sessió: 20 minuts
  - Freqüència: Una vegada al dia.
  - Duració de la fase: 5 dies a la setmana al llarg de tres setmanes
  - Progressió de la fase:

Dia 1-5: Moviments analítics de les articulacions involucrades en funció del nivell de lesió.

Dia 6-10: Estimulació sensitiva de les parts afectades (textures, formes, temperatura, vibració).

Dia 11-15: Interacció amb objectes i activitats funcionals.

Per tal de fer el seguiment de la intervenció, la investigadora principal de l’estudi es posarà en contacte amb el participant, via telefònica, una vegada a la setmana, per tal de resoldre possibles dubtes, aclariments o simplement per recordar la importància del seguiment de la intervenció. A més, l’aplicació generarà una notificació diària amb la finalitat de fomentar la continuïtat amb la intervenció.

**Etapa 3: Estudi fenomenològic:**

Paral·lelament a l’ECA, a mesura que els participants del grup experimental acabin la intervenció es realitzarà un estudi qualitatiu fenomenològic. Aquest tipus d’estudi ens permet descriure la vivència experimentada d’una persona a través d’entrevistes individuals semiestructurades^11^. A més, permet descriure un fenomen o una experiència a través de les pròpies persones que ho pateixen ^12^.

Els estudis quantitatius no valoren l’experiència de viure amb DMF i com aquest afecta la qualitat de vida de la persona. Una revisió sistemàtica del 2016 mostra que hi ha poca evidència científica qualitativa sobre la percepció que té la persona del DMF. Dins la evidència podem observar estudis que descriuen els factors psicològics derivats del DMF^13^ o l’afectació en la qualitat de vida de persones amb amputació de l’extremitat inferior^12^. Per tant, hi ha la necessitat de conèixer la vivència de les persones amb DMF després d’una amputació i identificar la satisfacció obtinguda amb la intervenció.

Aquest estudi començarà aproximadament a l’abril del 2022 i s’allargarà aproximadament fins al juny del 2023. Durant el procediment de l’estudi s’explicarà, via presencial o telefònica, l’estudi qualitatiu a aquelles persones que formin part del grup intervenció. Se’ls explicarà l’objectiu de l’estudi i la seva metodologia. Aquelles persones que acceptin participar se’ls enviarà, via email, el full de consentiment informat i la fulla d’informació. El número adequat de persones a participar és de 15. Aquest número està basat en la evidència científica considerat adequat tenint en compte el propòsit de la investigació, l’objectiu de l’anàlisi, el temps i els recursos disponibles^12^. Tot i així, és un pronòstic, serà la saturació de dades que ens indicarà el número de participants necessaris^14^. Un cop acabada la intervenció es deixarà triar a cada persona en quin horari i format (online, o presencial) li va millor realitzar la entrevista. Es ressaltarà la importància de realitzar l’entrevista presencialment per la importància de la comunicació no verbal tant de l’entrevistador/a com de l’entrevistat/da durant l’entrevista. L’entrevista seguirà una estructura semiestructurada on es parteix d’un guió però es va adaptant en funció de com avança la entrevista. Estarà formada per les següents parts: detalls demogràfics, aspectes relacionats amb l’amputació, experiència de patir DMF i identificació de la satisfacció obtinguda amb la intervenció. El temps aproximat per a cada entrevista serà de 30-45 minuts.

L’investigador que realitzarà l’entrevista serà una persona externa que no hagi realitzat la intervenció ni la aleatorització, per tal de no influir en les preguntes i respostes. Aquesta persona té sensibilitat teòrica sobre el DMF i sobre la teràpia mirall i això facilitarà poder profunditzar en els temes durant les entrevistes i entendre millor l’experiència viscuda. Posteriorment, dos membres del grup investigador realitzaran les transcripcions i anàlisis dels resultats. A través d’aquesta triangulació es pretén aconseguir la màxima objectivitat possible en els resultats. Les entrevistes seran gravades en format veu i vídeo per tal de poder realitzar una transcripció el més òptima possible i ajudarà a no perdre les expressions facials i corporals del/la participant. En el material enregistrat només i tindrà accés el grup investigador i l’investigador el qual hagi realitzat les entrevistes. Prèviament cada participant haurà firmat el consentiment de dret d’imatge i veu. Un cop acabat l’estudi, les gravacions seran eliminades. Un cop realitzades les transcripcions s’enviaran a cada participant per tal de corroborar la informació. Aquest procés també es realitzarà després de l’anàlisi dels resultats. Es pot observar la fulla de consentiment i la plantilla d’entrevista als annexes.

1. **Variables:**

Dues persones externes a la intervenció i a l’estudi fenomenològic realitzaran les valoracions. Aquestes persones seran dos/dues fisioterapeutes amb experiència clínica en l’àmbit de la neurorehabilitació i/o dolor, formats en mètodes de recerca, calibrats entre ells/elles per tal de minimitzar els biaixos i estaran cegats/des amb l’assignació del grups. Es realitzarà una valoració inicial, una valoració postintervenció (9 setmanes) i una valoració de seguiment (12 setmanes postintervenció). Les valoracions es realitzaran en els domicilis dels/les participants, en format presencial i tindran una durada aproximada de 25-30 minuts.

Les variables a estudiar les diferenciem entre variables sociodemogràfiques, característiques de l’amputació, variables independents i variables dependents. La primera valoració serà la que tindrà una duració més llarga ja que es recolliran totes les variables, però la segona i tercera valoració tindran una durada aproximada de 30 minuts ja que només es recolliran les variables dependents.

- **Variables sociodemogràfiques**; gènere, edat, estat civil, estatura, nivell d’estudis, nivell d’activitat física, antecedents patològics i traumàtics, fàrmacs administrats i dosis.

La recollida d’aquestes variables permetrà descriure la mostra de l’estudi i identificar que els/les participants compleixen els criteris d’inclusió mencionats anteriorment. D’aquesta forma, s’aconsegueix una mostra el més homogènia possible.

- **Característiques de l’amputació:** Causa de l’amputació, nivell d’amputació, costat, temps des de l’amputació, portador de pròtesis, número d’hores que utilitza la pròtesis.
- **Variables independents**: Tractament control i intervenció. Es recollirà tota la informació pertinent al tractament convencional de cada un dels/les participants.
- **Variables dependents:**
  - Dolor del membre fantasma: Short form McGill Pain Questionnaire. És una escala que valora el dolor des del punt de vista quantitatiu i qualitatiu. Està constituïda per 15 descriptors de dolor, dels quals 11 són categories sensitives i 4 afectives. A més a més, conté una escala visual analògica de dolor. És autocontestable i cada descriptor té tres columnes (mig, moderat, sever), on el/la propi/a participant ha de marcar el grau que persisteix aquell descriptor en el seu dolor. En cas de no ser present pot deixar-lo en blanc. Les columnes estan categoritzades sent 0; no dolor, 1; mig, 2; moderat, 3;sever. Es requereix entre 2-5 minuts a ser contestada.
  - Qualitat de vida: EuroQol-5D-5L^15^. És una escala autocontestable formada per 5 dimensions: mobilitat, autocura, activitats habituals, dolor/malestar i ansietat/depressió. Cada dimensió té 5 nivells: sense problemes, problemes lleus, moderats, greus i extrems. Se li demana al/la participant que indiqui el seu estat de salut marcant la casella junt amb la declaració més apropiada en cada una de les 5 dimensions. A més, conté una escala numèrica de 0 a 100 per quantificar el grau d’estat de salut el dia de la valoració. Es requereix entre 5-7 minuts a ser contestada.
  - Funcionalitat: Escala d’independència funcional (FIM)^16^. És una escala construïda a partir de 18 ítems dins de 6 àrees de funcionament: cuidat personal, control d’esfínters, mobilitat, marxa, comunicació i coneixement social. La màxima puntuació de cada ítem és 7 i la mínima de 1. Es requereix 5 minuts a ser contestada.
  - Aspectes psicològics: Beck depression inventory^17^. És una escala autocontestable de 21 preguntes. Cada pregunta està quantificada en una escala de 4 punts que va de 0 a 3, sent 0 que no experimenta símptomes i 3 experimenta símptomes de forma severa. Es requereix entre 5-10min a ser contestada.

A continuació, en forma de cronograma es detalla en quin moment serà recollida cada una de les variables exposades anteriorment.

| **Variable** | **Escala** | **Baseline** | **Post-intervenció (8 setmanes)** | **Follow-up**  **(12 mesos post- intervenció)** |
| --- | --- | --- | --- | --- |
| Sociodemogràfiques |  |  |  |  |
| Característiques de l’amputació |  |  |  |  |
| Dolor | SF-MPQ |  |  |  |
| Qualitat de vida | EuroQol-5D-5L |  |  |  |
| Funcionalitat | FIM |  |  |  |
| Aspectes psicològics | Beck |  |  |  |

La medicació serà una variable recollida des de dues setmanes abans de començar la intervenció fins a acabar-la.

1. **Anàlisi estadístic:**

Al realitzar un assaig cínic aleatoritzat amb metodologia mixta trobem dos anàlisis de dades diferenciats.

Anàlisis quantitatiu:

Les dades obtingudes com a variables de l’estudi seran codificades al acabar la recollida, processades i analitzades pels membres del grup de recerca.

La població que s’utilitzarà com a mostra en l’anàlisi inclourà a tots els/les participants que compleixin tots els criteris d’inclusió, no presentin cap criteri d’exclusió i hagin donat el seu consentiment per a participar en l’estudi. En cas d’abandonament d’un/a participant abans de completar l’estudi aquest/a no serà en cap cas substituït per un altre/a participant.

En el cas de participants que no hagin completat l’estudi per abandonament, no hagin complert íntegrament el tractament o inclús s’hagin canviat el tractament, les dades que s’hagin pogut recollir s’analitzaran per intenció de tractar. El mínim de sessions que han de realitzar per considerar els resultats serà del 80%.

El programa que s’utilitzarà per l’anàlisi estadístic serà el programa SPSS5. Es considerarà un nivell de confiança del 95%, i/o una significació estadística p<0,05.

Si durant el procés d’anàlisi de les dades es considera precís una desviació del mètode previst, es documentarà plenament les raons de qualsevol canvi.

Per a descriure la mostra es realitzarà una estadística descriptiva analitzant la mitjana, desviació estàndard, mínim, màxim i percentatges de les variables sociodemogràfiques. A més, es podrà mirar la mitjana dels/les inclosos/es i exclosos/es per tal de justificar les persones excloses. D’aquesta forma es podrà descriure la mostra i observar la homogeneïtat entre grups.

Seguidament, es realitzarà un anàlisis estadístic univariant i bivariant. Primer de tot es valorarà si l’estudi segueix o no una distribució normal en cada una de les variables. Aquest anàlisis permetrà determinar si utilitzar proves paramètriques o no paramètriques en l’anàlisi dels resultats en funció de la “p” valor.

A continuació s’analitzarà la igualtat de variàncies a través del test de Levenne i finalment es realitzarà l’anàlisi dels resultats. Es realitzarà un anàlisi pre i post intervenció entre grups i intragrups amb un seguiment a tres mesos. També podrem analitzar possibles correlacions entre variables.

Anàlisi qualitatiu:

L’anàlisi qualitatiu és un procés no lineal, interactiu i dinàmic. Estarà format per diferents parts seguint el mètode Colaizzi’s 7-stage method^18^. Es començarà per la transcripció de les entrevistes i familiarització amb les dades, seguidament es codificaran aquelles idees que estiguin relacionades amb la pregunta d’investigació. Els codis obtinguts s’agruparan en subcategories que comparteixin aspectes comuns i seran descrites i ubicades en el fenomen estudiat. Finalment, es categoritzarant aquestes subcategories buscant les idees generals i possibles relacions. L’anàlisi es realitzarà a través del programa Atlas.ti 8.0.

1. **Aspectes ètics**

**Recomanacions de Bona Pràctica Clínica:** Aquest estudi es base en les recomanacions de Bona Pràctica Clínica, a la Declaració de Helsinki de la Associació Mèdica Mundial (esmentada en la 64ª Assemblea General, Fortalesa, Brasil, Octubre 2013) i a la normativa legal aplicables. Per tant, tots els investigadors involucrats firmaran un certificat d’haver llegit i entès aquesta declaració. En cas necessari, s’incorporarà als annexes un formulari de notificació d’esdeveniments adversos. Es necessari mantenir un control rigorós i continu de la qualitat, que pugui garantir l’exactitud i el rigor científic de les dades obtingudes, mantenint les condicions de homogeneïtat durant el procés de recollida de la informació. En cas necessari, la creació d’un comitè logístic permetrà la coordinació adequada de tots els grups d’estudi i treball per contrastar la coordinació científica, l’assessorament metodològic i la qualitat de la informació obtinguda.

**Informació que serà proporcionada als participants i tipus de consentiments que serà sol·licitat en l’estudi:** el model d’informació que serà proporcionada als participants i el tipus de consentiment informat que es sol·licitarà seran especificats en els annexes al efecte. Així mateix, en els annexes constarà, en cas necessari, un full de renúncia per permetre als participants abandonar l’estudi en qualsevol moment. Tots els participants seran verbalment informats durant el procés d’inclusió en l’estudi per part d’un dels investigadors i se’ls enviarà via online les fulles d’informació amb tots els aspectes detallats. Posteriorment, els serà sol·licitat el consentiment informat. També se’ls proporcionarà el full de consentiment sobre drets d’imatge i veu.

**Confidencialitat:** en tot moment s’han de mantenir les normes més estrictes de conducta professional i confidencialitat, i el compliment de Reglament 2016/679 del Parlament Europeu i del Consell de 27 d’abril de 2016 relatiu a la protecció de les persones físiques (RGPD) i llei orgànica 3/2018, de 5 de desembre, de Protecció de Dades Personals i Garantía dels Drets Digitals (LOPD-GDD). El dret del participant a la confidencialitat és primordial. La identitat del participant en els documents de l’estudi ha de ser codificada, i únicament les persones autoritzades tindran accés a detalls personals identificables en el cas en què els procediments de verificació de dades exigeixin la inspecció d’aquests detalls. Els detalls personals identificables s’han de mantenir sempre confidencials i únicament tindran accés a ells l’investigador principal, el promotor i les persones autoritzades per aquest i les Autoritats Sanitàries corresponents. En relació a l’estudi fenomenològic, no es podrà codificar la identitat dels/les participants ja que durant la fase de transcripció tindrem el vídeo del/la participant. La persona responsable de realitzar les transcripcions garantitzarà en tot moment la confidencialitat de les dades i un cop realitzades les transcripcions els vídeos seran eliminats.

Podem observar tots aquests documents en els annexes.

1. **Grup d’investigació:**

- Fisioterapeuta especialitzada en neurorehabilitació i investigació traslacional en fisioteràpia (Sandra Rierola Fochs), que serà la investigadora principal i s’encarregarà de: dirigir l’estudi, la realització de la revisió sistemàtica, el disseny del protocol d’intervenció, l’aplicació del mètode Delphi, de l’estudi d’intervenció (mixte) i de l’anàlisi estadístic.
- Dos professors doctors de la UVic-UCC (Eduard Minobes Molina i Jose Antonio Merchán Baeza) que s’encarregaran de la supervisió del projecte.

1. **Experiència del grup d’investigació:**

Publicació del Dr. Jose Antonio Merchán Baeza en relació a la teràpia mirall.

Pérez-Cruzado D, Merchán-Baeza JA, González-Sánchez M, Cuesta-Vargas A. Systematic review of mirror therapy compared with conventional rehabilitation in upper extremity function in stroke survivors. Aust Occup Ther J. 2017;64(2):91-112.

Rierola-Fochs S, Varela-Vásquez LA, Merchán-Baeza JA, Minobes-Molina E. Development and validation of a graded motor imagery intervention for phantom limb pain in patients with amputations (GraMI protocol): A Delphi study. Int.J.Environ.Res.Públic.Health.2021;18:1-12.

1. **Beneficis esperats**

A nivell dels/les participants s’espera que el protocol GraMI disminueixi el DMF i com a conseqüència millori la qualitat de vida i disminueixin els factors psicològics associats.

A nivell dels hospitals participants s’espera que puguin conèixer una forma estandarditzada d’aplicació de la IMG en aquest perfil de pacients.

A nivell del grup investigador, s’espera realitzar comunicacions en congressos i publicacions sobre el protocol i els resultats obtinguts, així com la seva divulgació i instauració. A més, es pretén avançar en la tesis doctoral i intentar aconseguir projectes finançats per a seguir investigant en aquest àmbit.

1. **Possibles efectes no desitjats o secundaris**

La intervenció no és invasiva, per aquest fet no s’esperen efectes secundaris greus amb la intervenció realitzada.

1. **Contraprestació o assegurança per als participants**

No existeix cap contraprestació ni assegurança per als/les participants. Si aquests/es ho desitgen es podran elaborar informes d’evolució, comparant l’estat inicial, final i de seguiment de cada participant.

**Fulla d’informació per als participants:**

Alguns dels membres de l’equip d’investigació Research Group on Methodology, Methods, Models and Outcomes of Health and Social Sciences (M3O) de la Universitat de Vic-Universitat Central de Catalunya (UVic-UCC), portem a terme el projecte d’investigació “Efectes de la imatge motora graduada en el dolor del membre fantasma en el pacient amputat”. La investigadora principal d’aquest projecte és la Sandra Rierola Fochs.

En primer lloc, el grup investigador ha elaborat un protocol d’intervenció de la imatge motora graduada en el pacient amputat. Aquest protocol ha estat validat per un comitè d’experts en el tema, arribant a la obtenció del protocol final (protocol GraMI). En segon lloc, es busca valorar l’efectivitat del protocol obtingut. Per aquest fet, és pertinent realitzar un estudi d’intervenció. En el projecte participen els següents centres: Universitat de Vic, Hospital de la Santa Creu de Vic (falta detallar centres).

Els/les participants de l’estudi es dividiran en dos grups de forma aleatòria, a un grup li direm control i l’altre grup li direm intervenció. La intervenció per als dos grups tindrà una durada de 9 setmanes. Els/les participants que formin part del grup control realitzaran el tractament que es realitzi de forma protocol·lària en el centre/hospital, mentre que, els/les participants que formin part del grup intervenció realitzaran el protocol GraMI. Durant aquest temps es faran tres valoracions, una abans de començar el tractament, la segona després d’acabar el tractament (9 setmanes) i la tercera 12 setmanes després de la intervenció (seguiment). Les variables a valorar són el dolor del membre fantasma, la qualitat de vida, la funcionalitat i els aspectes psicològics. Es valorarà a través d’unes escales validades. A més, en els participants assignats en el grup intervenció se’ls oferirà participar en un altre estudi que consistirà en una entrevista individual semiestructurades per tal de comprendre com afecta el DMF en la qualitat de vida de les persones que el pateixen i identificar la satisfacció obtinguda amb la intervenció. Les entrevistes seran gravades de vídeo i veu per a un millor anàlisi posterior i tindran una durada aproximada entre 30-45 minuts. Un cop acabat l’estudi les gravacions seran eliminades.

Els beneficis esperats amb la seva participació en l’estudi són una possible disminució del dolor de membre fantasma, comportant a una millora de la qualitat de vida.

No hi ha riscos per a la seva participació.

En el context d’aquesta investigació li sol·licitem la seva col·laboració per incloure-ho en el grup de persones que participaran en el projecte, ja que vostè compleix amb els següents criteris d’inclusió:

- Adult major de 18 anys.
- Amputació d’una extremitat.
- Mínim de dolor de 3 en l’escala visual analògica del dolor.
- Farmacològicament estables
- Disposi de l’alta hospitalària.

Aquesta col·laboració es voluntària, no té cost per als participants i implica cooperar en:

- 3 sessions educatives de 30 minuts cada sessió on s’explicarà el procediment de cada una de les fases de l’intervenció i es podrà resoldre possibles dubtes. Cada dues setmanes es realitzarà una sessió (online/presencial)
- Realització d’una intervenció de 10-15 minuts, dos cops al dia, 5 dies a la setmana (cap de setmana de descans), al llarg de 9 setmanes.
- Es realitzaran 3 valoracions durant l’estudi, una valoració inicial al començar, una segona valoració a la finalització de la intervenció (9 setmanes), i una tercera i última valoració als tres mesos d’haver acabat la intervenció.

Tots els/les participants tindran assignat un codi pel qual és impossible identificar el/la participant amb les respostes donades, garantint totalment la confidencialitat. Les dades que s’obtindran de la seva participació no s’utilitzaran amb altra finalitat diferent de l’explicada en aquesta fulla informativa i passaran a formar part d’un fitxer de dades del qual serà responsable la investigadora principal i només i tindrà accés ella, i els altres membres de la investigació, Eduard Minobes Molina i Jose Antonio Merchán Baeza.

A l’acabar l’estudi, vostè podrà si ho desitja, sol·licitar a la investigadora principal la informació sobre els resultats, així com també rebrà un informe inicial i final de les valoracions que se li han realitzat.

El fitxer de dades de l’estudi estarà sota la responsabilitat de la investigadora principal, davant del qual podrà exercir en tot moment els drets que estableix la Llei orgànica 3/2018, del 5 de desembre, de Protecció de Dades Personals i garantia dels drets digitals i el Reglament general (UE) 2016/679, del 27 d’abril de 2016, de protecció de dades i normativa complementària.

Ens posem a la seva disposició per resoldre qualsevol dubte. Pot contactar amb la investigadora principal a través de l’adreça de correu electrònic: [sandra.rierola@uvic.cat](mailto:sandra.rierola@uvic.cat)

**Full de consentiment informat:**

Jo, (nom i cognoms) _____________________________________________, major d’edat, amb DNI ___________________, actuant en nom i interès propi.

**DECLARO QUE:**

He rebut la informació sobre el projecte “Efectes de la imatge motora graduada en el dolor del membre fantasma en el pacient amputat”, del qual se m’ha proporcionat el full informatiu annex a aquest consentiment i pel qual sol·licita la meva participació. He entès el significat, se m’han aclarit els dubtes i m’han explicat les accions que es deriven de l’estudi. Se m’ha informat de tots els aspectes relacionats amb la confidencialitat i protecció de dades per al que fa la gestió de dades personals que comporta el projecte i les garanties donades en compliment de la Llei orgànica 3/2018, del 5 de desembre, de protecció de dades personals i garantía dels drets digitals i el Reglament general (UE) 2016/679, del 27 d’abril de 2016, de protecció de dades i normativa complementària.

La meva col·laboració en el projecte és totalment voluntària i tinc dret a renunciar-hi en qualsevol moment, i revocar aquest consentiment, sense que això pugui influir negativament per a mi. En cas de retirar-me, tinc dret a que les meves dades siguin cancel·lades del fitxer de l’estudi.

Per tot això,

**DONO EL MEU CONSENTIMENT A:**

1. Participar en el projecte "Efectes de la imatge motora graduada en el dolor del membre fantasma en el pacient amputat”
2. Que l'equip de recerca Research Group on Methodology, Methods, Models and Outcomes of Health and Social Sciences (M3O) i la investigadora Sandra Rierola Fochs com IP, puguin gestionar les meves dades personals i difondre la informació que el projecte vagi generar. Es garanteix que es preservarà en tot moment la meva identitat i intimitat, amb les garanties establertes en la Llei orgànica 3/2018, de el 5 de desembre, de Protecció de Dades Personals i garantia dels drets digitals i el Reglament general (UE) 2016 / 679, de 27 de el 27 d'abril de 2016, de protecció de dades i normativa complementària.
3. Que l'equip Research Group on Methodology, Methods, Models and Outcomes of Health and Social Sciences (M3O), conservi tots els registres efectuats sobre la meva persona en suport electrònic, amb les garanties i els termes legalment previstos, pel temps que fos necessari per complir les funcions del projecte.

Ciutat, dia/mes/any

___________________________ ___________________________

Signatura del participant Signatura de l’IP

**FULLA INFORMATIVA DIRIGIDA ALS PARTICIPANTS SOBRE LA REALITZACIÓ D’ENTREVISTES INDIVIDUALS:**

Benvolgut participant,

Els investigadors que formen part del grup d’investigació en Metodología, Mètodes, Models i Resultats de Salut i Ciències Socials (M3O) de la Universitat de Vic- Universitat Central de Catalunya (UVic-UCC) porten a terme una investigació científica titulada “Efectes de la imatge motora graduada sobre el dolor del membre fantasma en el pacient amputat”.

La primera part (quantitativa) d’aquest projecte ja s’està desenvolupant. Per a la segona part (qualitativa), es vol comprendre com afecta el dolor del membre fantasma (DMF) en la qualitat de vida de les persones que el pateixen i identificar la satisfacció obtinguda amb la intervenció.

La realització d’entrevistes individuals es considera el millor mètode per a profunditzar en una experiència i comprendre un fenomen des de totes les seves perspectives, per tant, ens agradaria escoltar profundament la seva vivència i experiència.

Li oferim dues formes diferents de poder realitzar la entrevista, via Internet utilitzant un programa Zoom o de forma presencial. Es preveu que l’entrevista tingui una durada de 30-45 minuts aproximadament.

Desitgem que el/la participant tingui la flexibilitat per a decidir la data i hora en que pot estar disponible per a participar en l’entrevista. Per això, els/les investigadors/es que participaran en la entrevista intentaran en la mesura que sigui possible adequar el seu horari d’acord a la seva disponibilitat.

Durant l’entrevista, en el cas que sigui via Internet, li recomanem que estigui sol/a en una habitació o en un despatx sense distraccions com, per exemple, converses d’altres persones, soroll ambiental excessiu, etc. Els/les investigadors/es també estaran sols/es en una habitació o en un despatx per assegurar-se de que ningú més pugui escoltar l’entrevista o utilitzaran auriculars quan aquesta condició no es pugui complir durant l’horari laboral.

Per dur a terme l’anàlisi de dades d’aquesta investigació, necessitem anotar tot el que es parla durant l’entrevista, per aquesta raó, necessitem gravar la sessió per després poder transcriure tota l’entrevista paraula per paraula. Una vegada que s’hagi transcrit tot el contingut, se li enviarà per correu electrònic la seva gravació i la transcripció escrita i se li demanarà que confirmi la veracitat de la mateixa.

Una vegada que es confirmi la veracitat de la transcripció, la gravació s’eliminarà de forma permanent. El text de la conversació s’emmagatzemarà en una carpeta protegida amb una contrasenya i no serà accessible a tercers.

Si desitja fer alguna pregunta o necessita qualsevol aclariment relacionat amb la realització de l’entrevista, no dubti en posar-se en contacte amb la investigadora responsable de les entrevistes Sandra Rierola (630841957, sandra.rierola@uvic.cat).

Una vegada més, li agraïm la seva contribució a aquesta investigació científica com a participant.

Atentament.

**CONSENTIMENT PER A LA GRAVACIÓ EN SOPORT AUDIOVISUAL**

Persona que informa: Sandra Rierola Fochs

Participant:

**CONSENTIMENT**: Manifesto que estic d’acord amb:

Accepto lliurament a que sem gravi en suport audiovisual.

Entenc i comprenc que la gravació està relacionada a promoure el coneixement sobre el dolor del membre fantasma i identificar la satisfacció obtingut amb la intervenció. El seu us serà exclusiu en investigació, guardant l’anonimat. (Llei Orgànica 15/1999 de 13 de desembre, de protecció de Dades de Caràcter Personal, el Real Decret 994/1999, de 11 de juny). Es restringirà l’accés a les dades al personal autoritzat. L’emmagatzematge de la gravació complirà totes les mesures de seguretat oportunes per complir la llei citada.

Comprenc que puc retirar el meu consentiment en qualsevol moment, i que aquest no afectarà al tractament que rebi fins al moment o en el futur.

Firma del participant:

Data:

Firma de l’informador:

Data:

**FULLA DE RENÚNCIA**

**Títol de l’estudi:** Eficàcia de la imatge motora graduada en el dolor del membre fantasma en el pacient amputat

*Jo, ..................................................................................................................................., amb el D.N.I. nº ......................................, declaro que el meu desig és abandonar l’estudi en el qual he estat participant.*

*Declaro que no hi havia cap pressió ni insistència a donar les meves raons personals per la renúncia, d’acord amb les normes i els procediments d’inclusió en l’estudi.*

*Hi he parlat amb la: Sandra Rierola Fochs*

*Entenc que la meva participació era en tot moment voluntària i és la meva decisió abandonar l’estudi.*

*Comprenc que puc retirar-me de l’estudi:*

*1° Sense haver de donar cap explicació, i*

*2° Sense que aquest fet pugui repercutir en la meva relació amb la investigadora de l’estudi ni els promotors.*

*Així doncs, renuncio a seguir participant en aquest estudi..*

*Firmat:*

*Nom i cognoms de la participant:……………...…………………………………………..*

*D.N.I.: …………………………… Edat:………….. Data: ………………..*

*Firma del/de la investigador/a principal:*

*Investigador/a principal:* Sandra Rierola Fochs

*Data: ………………..*

**Guia entrevista semiestructurada:**

Nº del/la participant:

Data: ........./........../...........

Edat:

Gènere:

Característiques de l’amputació:

| Nivell | Costat | Causa | Temps d’evolució | Pròtesis (tipus) |
| --- | --- | --- | --- | --- |
|  |  |  |  |  |

| Pregunta general | Preguntes alternatives (per profunditzar la pregunta general) |
| --- | --- |
| Vivència del dolor | |
| 1. Quan et va aparèixer el dolor? | - Va aparèixer de forma progressiva o de cop? - Fluctuava durant el dia o sempre era igual? - Tenies dolor previ a l’amputació? |
| 1. Què vas pensar en el moment que vas començar a tenir dolor? | - Previ a l’amputació t’havien explicat que podia aparèixer aquest dolor? - Va haver-hi un període de temps que no vas comentar a ningú que tenies el dolor? En cas afirmatiu quin va ser el motiu? - Quina és la primera persona amb la qual és parlat sobre aquest dolor? Perquè amb aquesta persona? |
| 1. En el moment d’expressar-ho algú et va explicar què era aquell dolor i perquè el tenies? | - Si: Què vas sentir en aquell moment? - No: T’hauria agradat que algú t’ho hagués explicat? - No: Com va ser la sensació de no saber què li passava al teu cos? |
| 1. Com descriuries el dolor quan et va aparèixer? | - Ha canviat amb el temps o segueixes tenint els mateixos símptomes? - L’aparició del dolor canvia quan tens diferents emocions com estar feliç, estressat o trist? |
| 1. Ha influenciat en la teva vida diària el fet de viure amb dolor? | - Si: En quins aspectes? Actualment creus que el dolor és un condicionant a la hora de prendre decisions en la teva vida diària? Perquè? - Hi ha alguna activitat que abans feies però ara no pots per el dolor? |
| 1. Coneixes altres persones que també tenen dolor després de l’amputació? | - Si: T’ha ajudat en algun aspecte? - No: Creus que t’hauria anat bé conèixer algú que es troba en la mateixa situació? |
| 1. M’expliques la teva forma de manejar el dolor? (tant mèdica com psicològicament) |  |
| Satisfacció amb la intervenció | |
| 1. Havies realitzat tractaments específics previs pel dolor? | - Quina és la teva experiència amb aquests tractaments? - Vas notar canvis en el dolor? *Si: quins canvis has notat?* - Va canviar la teva qualitat de vida? *Si: en quins aspectes t’ha canviat la qualitat de vida?* |
| 1. Com era el teu dolor quan vas començar a realitzar la intervenció? |  |
| 1. Van ser d’utilitat les sessions educatives prèvies a cada una de les fases? | - Si: Per què? - No: Per què |
| 1. Ha estat difícil realitzar la intervenció? *Si: quines són les dificultats que has experimentat?* | - Com ha estat l’acompanyament que has tingut durant la intervenció? - Com era l’ambient que tenies durant la intervenció? (espai, soroll, el fet d’estar sola..). |
| 1. Què esperaves de la intervenció abans de començar? | - S’han complert aquests objectius? |
| 1. Com ha evolucionat el dolor durant aquestes setmanes? | - Segons la teva opinió, quin ha estat el rol de la intervenció en la modificació del dolor? Creus que hi ha hagut altres factors que hi ha pogut influenciar? |
| 1. Quins canvis ha experimentat en el seu dia a dia amb la intervenció? |  |
| 1. Recomanaries aquesta intervenció a una persona amb el mateix dolor? | - Si: Quins punts forts explicaries de la intervenció en aquesta persona?   No: Perquè? |

**Referències bibliogràfiques**

1. Aiyer R, Barkin RL, Bhatia A, Gungor S. A systematic review on the treatment of phantom limb pain with spinal cord stimulation [Internet]. Vol. 7, Pain management. Future Medicine Ltd London, UK ; 2017 [cited 2021 May 10]. p. 59–69.

2. Fehlings MG, Sarhane KA, Wilson J, Tsao JW, Sb F, Bn P, et al. a randomized, Controlled Trial of Mirror Therapy for Upper Extremity Phantom limb Pain in Male amputees. 2017 [cited 2021 May 10];8.

3. Collins KL, Russell HG, Schumacher PJ, Robinson-Freeman KE, O’Conor EC, Gibney KD, et al. A review of current theories and treatments for phantom limb pain. J Clin Invest. 2018;128(6):2168–76.

4. Vannuccini S, Petraglia F. Recent advances in understanding and managing phantom limb pain. F1000Research. 2019;8:1–11.

5. Mg DF, Manual T, Fisioterapeuta O. Imaginería motora graduada en el síndrome de miembro fantasma con dolor. 2012;19(4):209–16.

6. Osinski T. Imaginería motora graduada. Colloids Surfaces A Physicochem Eng Asp. 2019;39(2):1–10.

7. Ebrahimi N, Rojhani-Shirazi Z, Yoosefinejad AK, Nami M. The effects of virtual reality training on clinical indices and brain mapping of women with patellofemoral pain: a randomized clinical trial. BMC Musculoskelet Disord [Internet]. 2021;22(1):1–10.

8. Moher D, Hopewell S, Schulz KF, Montori V, Gøtzsche PC, Devereaux PJ, et al. CONSORT 2010 explanation and elaboration: Updated guidelines for reporting parallel group randomised trials. Int J Surg [Internet]. 2012;10(1):28–55.

9. O’Brien BC, Harris IB, Beckman TJ, Reed DA, Cook DA. Standards for reporting qualitative research: A synthesis of recommendations. Acad Med. 2014;89(9):1245–51.

10. Osumi M, Ichinose A, Sumitani M, Wake N, Sano Y, Yozu A, et al. Restoring movement representation and alleviating phantom limb pain through short-term neurorehabilitation with a virtual reality system. Eur J Pain (United Kingdom). 2017;21(1):140–7.

11. Lambert C. Edmund Husserl: la idea de la fenomenología. Teol y vida. 2006;47(4):517–30.

12. Trevelyan EG, Turner WA, Robinson N. Perceptions of phantom limb pain in lower limb amputees and its effect on quality of life: A qualitative study. Br J Pain [Internet]. 2016;10(2):70–7.

13. Roșca AC, Baciu CC, Burtăverde V, Mateizer A. Psychological Consequences in Patients With Amputation of a Limb. An Interpretative-Phenomenological Analysis. Front Psychol. 2021;12(May).

14. Nuevos avances en la investigación social. La investigación social de segundo orden. 1998;

15. Herdman M, Gudex C, Lloyd A, Janssen M, Kind P, Parkin D, et al. Development and preliminary testing of the new five-level version of EQ-5D (EQ-5D-5L). Qual Life Res. 2011;20(10):1727–36.

16. de Azevedo Alves GA, Martinez BP, Lunardi AC. Assessment of the measurement properties of the Brazilian versions of the Functional Status Score for the ICU and the Functional Independence Measure in critically ill patients in the intensive care unit. Rev Bras Ter Intensiva. 2019;31(4):521–8.

17. Beck AT, Ward CH, Mendelson M, Mock J, Erbaugh J. An Inventory for Measuring Depression, Archives of General Psychiatry, 4. Arch Gen Psychiatry. 1961;4(6):561–71.

18. Wirihana L, Welch A, Williamson M, Christensen M, Bakon S, Craft J. Using Colaizzi’s method of data analysis to explore the experiences of nurse academics teaching on satellite campuses. Nurse Res. 2018;25(4):30–4.

**Cronograma**

|  | 2021 | 2021 | | | | 2022 | | | |
| --- | --- | --- | --- | --- | --- | --- | --- | --- | --- |
|  | Octubre-Desembre | Gener-Març | Abril-Juny | Juliol-Setembre | Octubre-Desembre | Gener-Març | Abril-Juny | Juliol-Setembre | Octubre-Desembre |
| Disseny del protocol d’estudi |  |  |  |  |  |  |  |  |  |
| Disseny de l’aplicació |  |  |  |  |  |  |  |  |  |
| Reclutament de participants |  |  |  |  |  |  |  |  |  |
| Intervenció |  |  |  |  |  |  |  |  |  |
| Estudi qualitatiu |  |  |  |  |  |  |  |  |  |
| Anàlisi dels resultats |  |  |  |  |  |  |  |  |  |
| Divulgació dels resultats |  |  |  |  |  |  |  |  |  |
| Redacció de la tesis |  |  |  |  |  |  |  |  |  |
| Dipòsit de la tesis |  |  |  |  |  |  |  |  |  |
