# Supplementary material for: Effectiveness of graded motor imagery protocol in phantom limb pain in amputed patient: Protocol of a randomized clinical trial
Source: PLoS One. 2022 Aug 25;17(8):e0273356. doi: 10.1371/journal.pone.0273356 (PMC9409541; doi:10.1371/journal.pone.0273356)
Supplement: S4 File — (DOCX) [file pone.0273356.s004.docx]

**
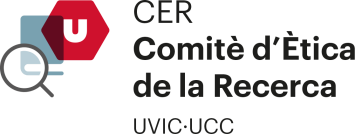
Favourable opinion report**

**Investigation project**

**Internal code: 185/2021**

**November 24, 2021**

PhD. Ester Busquets Alibés

Technical Secretary of the UVic-UCC Research Ethics Committee

**CERTIFIES**

That the CER of the UVic-UCC at the meeting on November 22, 2021, has evaluated the research project:

Title: Efficacy of graded motor imaging in phantom limb pain in amputated patients.

Internal code: 185/2021.

IP: Sandra Rierola Fochs.

Consider that:

- The necessary suitability requirements are met in relation to the objectives and the methodological design.
- Ethical requirements are met, both in obtaining informed consent and in aspects related to confidentiality.
- The competence of the IP and the available means are appropriate to develop the study

Therefore, the CER issues a **FAVORABLE OPINION**^1^.

Ester Busquets Alibés - DNI

Firmado digitalmente por Ester Busquets Alibés - DNI 33946176Q (TCAT)

33946176Q (TCAT) Fecha: 2021.11.24 00:19:09

+01'00'

24/11/2021

Signature of the technical secretary

^1^ A favourable opinion entails the following obligations for the principal investigator:

a) Submit, if necessary, the project to external calls (competitive or not) with the same essential elements that have been favourably evaluated by the CER.

b) Develop, if necessary, the project with the same essential elements that have been favourably evaluated by the CER.
